# NDST3 suppression restores lysosomal acidification and ameliorates amyloid-β and MAPT/tau pathology in Alzheimer’s disease

**DOI:** 10.1186/s40035-026-00549-1

**Published:** 2026-04-21

**Authors:** Chuanhua Ge, Kun Wang, Huiyuan Tang, Yiling Ke, Huai Wang, Qiang Fu, Yun Xiu, Yongzheng Guo, Yun-Fang Jia, Zhimin Long, Guiqiong He, Qing Tang

**Affiliations:** 1 Center for Neuroscience Research, School of Basic Medical Sciences, Chongqing Medical University, No.1 YiXueYuan Road, YuZhong District, Chongqing, 400016 China; 2 Department of Neurosurgery, Tongren People’s Hospital, Tongren, 554300 Guizhou China; 3 Key Laboratory of Major Brain Disease and Aging Research (Ministry of Education), Chongqing Medical University, No.1 YiXueYuan Road, YuZhong District, Chongqing, 400016 China; 4 Division of Cardiology, The First Affiliated Hospital of Chongqing Medical University, Chongqing, 400016 China; 5 Department of Physiology, School of Basic Medical Sciences, Chongqing Medical University, Chongqing, 400016 China; 6 Department of Anatomy, School of Basic Medical Sciences, Chongqing Medical University, Chongqing, 400016 China

**Keywords:** Alzheimer's disease, Amyloid plaque, Lysosomal acidification, MAPT/tau, N-deacetylase and N-sulfotransferase 3, Histone deacetylase 6

## Abstract

**Background:**

Impairment of lysosomal acidification has recently been identified as a critical driver of amyloid-β and MAPT/tau pathology in Alzheimer’s disease (AD). Restoring lysosomal acidification is a promising strategy for AD treatment. N-deacetylase and N-sulfotransferase 3 (NDST3) is a newly discovered tubulin deacetylase that regulates lysosomal acidification by influencing the recruitment of V-ATPase V1 subunits to lysosomes. Nevertheless, the role of NDST3 in AD remains entirely unexplored.

**Methods:**

We began by comparing the effects of NDST3 and histone deacetylase 6 (HDAC6), a well-known tubulin deacetylase with established roles in AD, on lysosomal acidification. Using HT22 cell-based models of AD, we knocked down NDST3 to examine its role in lysosomal acidification and degradative function in the context of this disease. We also evaluated the expression profile of NDST3 in both in vitro and in vivo models of AD. Finally, we investigated the consequences of NDST3 suppression on lysosomal acidity and related AD pathological features in the hippocampi of 3 × Tg-AD mice.

**Results:**

NDST3 differs from HDAC6 in the subcellular spatial patterns of catalyzing microtubule deacetylation but parallels HDAC6 in regulating lysosomal pH. In HT22 cells with APP695^Swe^ overexpression, knockdown of NDST3 lowered lysosomal pH by promoting the assembly of the V-ATPase holoenzyme on the lysosomal membrane and enhanced the autophagic degradation of aberrant Aβ and MAPT/tau. Notably, NDST3 levels were found to be elevated in the brains of AD models and patients. Reducing NDST3 expression in the hippocampi of 3 × Tg-AD mice facilitated lysosomal reacidification, which decreased the abnormal accumulation of amyloid plaques and MAPT/tau tangles, mitigated neuronal damage, and ameliorated cognitive deficits.

**Conclusions:**

Our study identified NDST3 as a key factor regulating lysosomal acidity in AD. Suppressing NDST3 restores lysosomal function in AD and protects against AD pathology, highlighting NDST3 as a promising therapeutic target for AD.

**Supplementary Information:**

The online version contains supplementary material available at 10.1186/s40035-026-00549-1.

## Background

Alzheimer’s disease (AD) is a typical neurodegenerative disorder characterized by progressive neuronal death and cognitive decline and remains a significant global health challenge [1]. The extremely complex pathogenesis of AD has impeded the development of therapeutic approaches. Currently, there are a few drugs that can slow the progression of AD, but none can cure the disease. Substantial challenges remain in the search for more effective therapeutic strategies for approximately 50 million people worldwide living with AD [2, 3]. The accumulation and deposition of toxic misfolded proteins, including amyloid-β (Aβ) and hyperphosphorylated microtubule-associated protein tau (MAPT)/tau, due to the failure of protein quality control within cells, are among the most recognized pathological changes that induce neurodegeneration in AD [4]. The presence of Aβ-containing plaques and MAPT/tau-containing neurofibrillary tangles (NFTs) triggers a cascade of downstream events, such as neuroinflammation and oxidative stress, that ultimately lead to neuronal death and related symptoms [5, 6]. Recent studies have additionally suggested that small soluble oligomers or protofibrils formed from Aβ and MAPT/tau are particularly toxic to neurons [7, 8]. Eliminating aberrant Aβ and MAPT/tau accumulation, along with their deposition, represents one of the most promising therapeutic strategies for AD [3].

Lysosomes are intracellular organelles that clear misfolded proteins and aggregates, including aberrant Aβ and MAPT/tau oligomers and plaques associated with AD, ensuring proper cellular homeostasis. Some lysosomal enzymes, such as glucocerebrosidase and cathepsin B (CTSB), are involved in Aβ autophagic degradation [9, 10]. Abnormally modified MAPT/tau is also degraded through the autophagy-lysosomal pathway [11]. Defects in the lysosomal system lead to failed clearance of these pathological proteins and aggregates in AD. Blockage of intraneuronal Aβ and MAPT/tau degradation, resulting from impaired lysosomal function, is widely recognized as the cause of senile plaque and NFT formation in AD [12–14]. In addition to Aβ and MAPT/tau themselves, specific proteins that contribute to Aβ production also undergo lysosomal degradation. The amount of amyloid-β precursor protein (APP), from which Aβ is produced, is rigorously controlled to maintain a steady pool of Aβ proteins. In neurons, full-length APP undergoes nonamyloidogenic cleavage mediated by α- and γ-secretases, amyloidogenic cleavage by β- and γ-secretases, or degradation via lysosomes [15]. Compromised lysosomal function not only drives increased amyloidogenic processing of APP, but also impedes APP degradation, resulting in increased Aβ formation and accumulation [16, 17]. Moreover, the APP-derived C-terminal fragment β (APP-CTFβ), a key intermediate in the amyloidogenic processing of APP, also undergoes lysosomal degradation [18]. APP-CTFβ serves as the direct precursor of Aβ, yielding Aβ following cleavage by the γ-secretase complex [5]. In light of these observations, proper lysosomal functionality is pivotal for mitigating Aβ and MAPT/tau pathology.

The acidic environment within lysosomes is essential for their resident enzymes to degrade lysosomal substrates. Impairment of lysosomal acidification has been recognized as an early indicator of neurodegeneration [18, 19]. In the context of AD, the mechanisms of lysosomal acidification are disrupted, leading to the alkalinization of lysosomes [20]. Faulty lysosomal acidification, followed by impaired lysosomal function, was reported to drive autophagic build-up of Aβ as well as the exacerbation of tauopathy in neurons [19, 21, 22]. Restoring a normal lysosomal pH guarantees the recovery of lysosomal degradation of such toxic proteins, which is a promising approach for the treatment of AD [19, 23].

Lysosomal acidification is driven primarily by vacuolar-type H^+^-ATPase (V-ATPase), a complex that resides on the lysosomal membrane and is responsible for the translocation of protons from the cytosol to the lysosomal lumen, thereby lowering the luminal pH [24]. The V-ATPase complex is composed of multiple subunits organized into two domains: the peripheral V1 domain and the lysosomal membrane-integral V0 domain. V-ATPase function depends on V1–V0 holoenzyme assembly on lysosomes [25, 26]. Microtubule acetylation and stabilization serve as a structural scaffold that facilitates the recruitment of V-ATPase V1 subunits to lysosomes in the perinuclear region, where V-ATPase exhibits higher activity compared to peripheral regions [27, 28]. Hyperacetylation of α-tubulin, induced by depletion of the microtubule deacetylase histone deacetylase 6 (HDAC6) or pharmacological inhibition with trichostatin A, has been reported to reduce lysosomal pH and restore lysosomal function in AD [29, 30]. Interestingly, we recently discovered a novel microtubule deacetylase, N-deacetylase and N-sulfotransferase 3 (NDST3), that regulates lysosomal acidification in RPE1 cells. Suppression of NDST3 promoted the spatial coupling of V-ATPase V1 subunits and lysosomes on acetylated microtubule tracks, restoring normal lysosomal pH in cells treated with the alkalinizing agent bafilomycin A1 (Baf A1) [27, 31].

Building on previous findings, in this study, we further investigated the regulatory role of NDST3 in lysosomal acidification in the context of AD. We first characterized the functional distinctions between NDST3 and HDAC6 in mediating microtubule deacetylation and lysosomal acidification. Subsequently, using postmortem human brain tissues, cell-based models, and transgenic mice, we evaluated whether targeted suppression of NDST3 could restore lysosomal pH homeostasis, promote the autophagic clearance of aberrant Aβ and MAPT/tau, and ultimately alleviate neurodegeneration and cognitive decline in AD. Through this work, we aim to uncover a previously unrecognized role of NDST3 in AD-associated lysosomal dysfunction, thus highlighting a promising therapeutic strategy for this disease.

## Materials and methods

### Animals and breeding strategy

3 × Tg-AD mice were purchased from Beijing HFK Bioscience Co., Ltd. *Ndst3* knockout mice were generated by Jiangsu GemPharmatech Co., Ltd. using CRISPR/Cas9 technology. 3 × Tg-AD mice and *ndst3* knockout mice (with the same genetic background) were crossed for several generations to produce 3 × Tg-*Ndst3*^+/-^ mice. Both male and female mice were used in this study. Mice of both sexes were randomly assigned to all experimental groups. The genotypes of all mice were identified by PCR experiments. The primers are listed in Table S1. According to the IACUC guidelines, the mice were housed in animal facilities with specific pathogen-free environment at Chongqing Medical University, with a 12-h light/dark cycle and a temperature of 22 ± 2 °C, where they had free access to food and water. All animal experimental procedures were approved by the Animal Care and Use Committee of Chongqing Medical University (approval No. IACUC-CQMU-2023-0123).

### Cell culture and treatments

HT22 mouse hippocampal neuronal cells (kindly provided by the Cell Bank, Chinese Academy of Sciences; Research Resource Identifier: CSTR:19375.09.3101MOUSCSP5419) and human retina pigmented epithelial (RPE1) cells (ATCC CRL-4000) were cultured at 37 °C in a humidified incubator with 5% CO_2_ in Dulbecco’s Modified Eagle’s Medium (DMEM) (#C11995500BT, Gibco, Waltham, MA) or DMEM/F-12 (#11320033, Gibco) containing 10% fetal bovine serum (#SX1101, Sorfa, Beijing, China), 100 U/mL penicillin, and 100 µg/mL streptomycin (#C0222, Beyotime, Shanghai, China). The cell lines had been confirmed as contamination-free by the provider and were routinely monitored for contamination in our laboratory. To generate *NDST3* and *HDAC6* knockout (KO) cells, RPE1 cells were infected with lentivirus carrying *Ndst3*-targeting sgRNA (ACAGCGTAGCCCATGTCCGT) and *Hdac6*-targeting sgRNA (TCCCTTGCAGTCCCACGATT), respectively. Wild-type (WT) cells received a control sgRNA (ACGGAGGCTAAGCGTCGCAA) not targeting any gene. Lipofectamine™ 2000 transfection reagent (#52887, Invitrogen, Carlsbad, CA) was used to transfect HT22 cells with plasmids expressing APP695^Swe^ or MAPT/tau^P301L^ (a gift from Zhifang Dong lab in the Children’s Hospital of Chongqing Medical University, Chongqing, China) to construct APP695^Swe^- or MAPT/tau^P301L^-overexpressing model cells. These cells were further transfected with an shRNA targeting *Ndst3* (target sequence: GCGCACACAAATCACAAATTT) or a scramble control shRNA (target sequence: CCTAAGGTTAAGTCGCCCTCG), both expressed from the pLKO.1 vector. NDST3-overexpressing (*Ndst3* OE) cells were generated using the same ORF plasmid described previously [27], with empty vector transfection as controls. The cells were used for subsequent experiments 48 h or 72 h after plasmid transfection.

### Lysosomal pH measurements in vitro

The absolute lysosomal pH value was measured using the previously described method [27, 32]. In brief, gradient pH standard buffers were prepared using MES solution containing 20 mM 2-[N-morpholino] ethanesulfonic acid, 110 mM KCl, and 20 mM NaCl. The experimental cells and the cells used for drawing the lysosomal pH standard curve were incubated with isotonic buffer (105 mM NaCl, 5 mM KCl, 6 mM HEPES-Acid, 4 mM HEPES-Na, 5 mM NaHCO_3_, 60 mM mannitol, 5 mM glucose, 0.5 mM MgCl_2_, 1.3 mM CaCl_2_, adjusted to pH 7.4) containing LysoSensor™ yellow/blue DND-160 (#L7545, Thermo Fisher Scientific, Waltham, MA) at room temperature. Specifically, RPE1 cells were incubated with 2 μM LysoSensor for 3 min, while HT22 cells were incubated with 1.5 μM LysoSensor for 4 min. Immediately after the LysoSensor incubation, the liquid containing LysoSensor was discarded, and the cells were quickly washed three times with isotonic buffer. The cells were then incubated at room temperature for 10 min in the gradient pH standard buffers in the presence of 10 μM monensin (#MZ2101, MKbio, Shanghai, China) and 30 μM nigericin (#MZ2157, MKbio). A plate reader (Tecan, Männedorf, Switzerland, Spark) was used to measure the fluorescence intensity of each well at dual excitation wavelengths, 340 nm (*F*_340 nm_) and 380 nm (*F*_380 nm_), with the emission wavelength set at 527 nm. The lysosomal pH value standard curve was generated based on the standard pH values and the measured fluorescence intensity ratios (*F*_340 nm_/*F*_380 nm_). The lysosomal pH of the experimental cells was finally calculated using the standard curve and the fluorescence intensity ratios.

### Cathepsin activity assay

Cells were plated in 3.5-cm confocal dishes and cultured until reaching 60%–70% confluency. Magic Red CTSB substrate MR-(RR)_2_ (#6134, ImmunoChemistry, Davis, CA) was diluted 1:10 with sterile water. The cell culture medium was removed, and a 1:25 dilution of Magic Red CTSB in cell culture medium was added to the confocal dish and incubated at 37 °C in the dark for 1 h. The medium with the probe was then removed, and the cells were washed with 1 × PBS (#C10010500BT, Gibco) for 2 × 1 min. Hoechst 33342 (#639, ImmunoChemistry) at a concentration of 1 µg/mL was used to stain the nuclei, with the cells incubated at 37 °C for 10 min. The medium was replaced with PBS, and the cells were immediately imaged using a confocal microscope (Leica, Wetzlar, Germany, SP8) with an excitation wavelength of 561 nm and an emission wavelength of > 610 nm. Fiji software was used to analyze the Magic Red intensity in each cell.

### Reverse-transcription quantitative PCR

Following the manufacturer’s protocol, TRIzol reagent (#DP424, Tiangen Biotechnology, Beijing, China) was used to isolate total RNA from cell samples. RNA was reverse-transcribed into cDNA using All-In-One 5 × RT MasterMix (#G592, Shanghai ABM Bioscience, Shanghai, China). Then, real-time PCR was performed using BlasTaq™ 2 × qPCR MasterMix (#G891, Shanghai ABM Bioscience) on the CFX Connect™ Real-Time PCR Detection System (Bio-Rad, Philadelphia, PA) to assess mRNA levels. Primers were designed based on the mouse gene sequence from NCBI GenBank (ncbi.nlm.nih.gov), and their specificity was pre-confirmed using the Primer-BLAST tool. The primer sequences were as follows: *Ndst3* (Forward: GGCTGCTTCTCTAGTCCCGAAAG; Reverse: CGCTGATGCTGATACCAGGAGTAC), *App* (Forward: GTTTGGCACTGCTCCTGCTG; Reverse: GCACCAGTTCTGGATGGTCA), *Mapt* (Forward: GGCCTGAAAGCTGAAGAAGC; Reverse: CTTCCAGTCCCGTCTTTGCT), *Gapdh* (Forward: GAAGGTCGGTGTGAACGGAT; Reverse: ACTGTGCCGTTGAATTTGCC), and *β-actin* (Forward: CAGCCTTCCTTCTTGGGTATG; Reverse: GGCATAGAGGTCTTTACGGATG), synthesized by Sangon Biotech (Shanghai, China). The relative mRNA levels were calculated using the 2^−ΔΔCt^ method, with normalization to *β-actin* or *Gapdh*.

### Dextran degradation assay

The dextran degradation assay was performed as previously described [27, 33]. In brief, cells were seeded in a 3.5-cm confocal dish. When 60%–70% confluency was reached, the cell culture medium was replaced with medium containing 0.4 mg/mL Alexa Fluor 647 (AF647)-dextran (#D22914, Invitrogen) and incubated at 37 °C for 20 min. The medium was aspirated, and cells were washed three times with 1 × PBS. Cells for uptake assay were immediately fixed with 4% PFA at room temperature for 15 min, while cells for tracking analysis were placed back in the incubator for further incubation for 4 h before fixation. After fixation, cells were washed three times with 1 × PBS, incubated with Hoechst 33342 at 37 °C for 10 min, and then washed again with 1 × PBS. Imaging was performed immediately using a confocal microscope (Leica, SP8) with the excitation/emission wavelengths recommended by the manufacturer. Fiji software was used to quantify the fluorescence intensity. The fluorescence intensity for uptake and tracking analyses was recorded as *F*_0_ and *F*_1_, respectively. The degradation rate of AF647-dextran was calculated using the following equation: Degradation (%) = (*F*_0 _− *F*_1_)/*F*_0_ × 100%.

### Enzyme-linked immunosorbent assay (ELISA)

Following relevant treatment, cells were centrifuged, and the supernatant was collected. The levels of Aβ40 and Aβ42 in the supernatant were detected using sandwich ELISA kits (#ADS-1743H1 and #ADS-1363H1, Jiangsu Aidisheng Biological Technology, Yancheng, China) according to the manufacturer’s instructions. Standards and samples were measured in duplicates, with sample concentrations diluted two-fold before loading. The absorbance at 405 nm was measured using a microplate reader (BioTek, Winooski, VT; ELX800).

### Lysosome isolation

Lysosomes were isolated using the Lysosome Enrichment Kit for Tissues and Cultured Cells (#89839, Thermo Fisher Scientific). In brief, the cells in a 15-cm dish were digested using trypsin, washed twice with 1 × PBS, and centrifuged at 500 × *g* for 5 min at 4 °C. The supernatant was discarded after centrifugation. The cell pellets were lysed in Reagent A containing 1 mM PSMF and 1 × protease inhibitor cocktail by Dounce homogenization, then neutralized with Reagent B. The samples were then centrifuged at 500 × *g* for 10 min at 4 °C, and the supernatant was collected and subjected to ultracentrifugation in discontinuous density gradient of OptiPrep Media at 145,000 × *g* for 2 h at 4 °C. Following ultracentrifugation, the lysosome-enriched fraction at the top of the density gradient was harvested. This fraction was then diluted with 3 volumes of 1 × PBS and centrifuged at 18,000 × *g* for 30 min at 4 °C to pellet the lysosomes. The resulting pellet was collected and subjected to a surface-wash with Gradient Dilution Buffer to remove residual contaminants. The lysosome pellet was saved for subsequent experiments.

### Cell survival assay

Cells in a 12-well plate at ~ 70% confluency were transfected with APP695^Swe^ or MAPT/tau^P301L^ combined with *Ndst3*-targeting shRNA or scramble shRNA. Blank control (no cells plated) and positive control (with shRNA treatment but without APP695^Swe^ or MAPT/tau^P301L^ treatment) wells were set up. Forty-eight hours after the transfection of shRNA, the cells were subjected to cell survival assay using calcein AM (#C2012, Beyotime) according to the manufacturer’s instructions. Briefly, the cell culture medium was removed from the wells of the plate. The cells were gently washed twice with 1 × PBS (containing MgCl_2_ and CaCl_2_). Then 500 μL of medium containing 3 μM calcein AM was added to each well and incubated at 37 °C for 30 min, protected from light. Fresh medium was then used to replace the calcein-containing medium, and the cells were incubated at 37 °C for another 30 min, followed by two gentle washes with 1 × PBS. Finally, serum-free medium was added into the wells and a plate reader (Tecan, Spark) was used to detect the fluorescence intensity at 485 nm/535 nm (excitation/emission) in the wells. A fluorescence microscope (Leica, DMI8 THUNDER) was also used to capture representative images of the cells. The fluorescence intensities of the experimental, blank control, and positive control wells were recorded as *F*_x_, *F*_0_, and *F*_max_, respectively. The percentage of cell viability in the experimental wells was calculated using the following equation: Cell survival (%) = (*F*_x _− *F*_0_)/(*F*_max _− *F*_0_) × 100%.

### Human tissue samples

Postmortem human brain tissue slices (paraffin-embedded) were obtained from the Human Brain Bank, Central South University Xiangya School of Medicine, with approval by the Institutional Review Board of School of Basic Medical Science, Central South University (approval No. 2020KT-37).

### Mouse tissue sample preparation for immunostaining and histological staining

For mouse brain tissue preparation, mice were euthanized via an overdose of inhaled isoflurane (#R510-22-2, RWD Life Science Co., Ltd, Shenzhen, China) followed by cervical dislocation. The brains were dissected from the mice, and the hippocampus was harvested, which was immediately flash-frozen in liquid nitrogen and stored in a − 80 °C freezer, or fixed in 4% PFA at 4 °C for at least 24 h, dehydrated through graded alcohol, embedded in paraffin, sectioned into 4-μm thick coronal slices with a paraffin slicer (Leica, RM2016), and stored at room temperature.

### Blood biochemical analyses

Blood samples were collected from mice via retro-orbital venous plexus puncture under light anesthesia via isoflurane inhalation (3%–4% for induction, 1%–2% for maintenance). The collected blood was placed in serum separation tubes and allowed to stand at room temperature for 30 min to promote coagulation. Subsequently, the samples were centrifuged at 3000 × *g* for 15 min at 4 °C. The serum was collected, aliquoted, and stored at − 80 °C without repeated freeze–thaw cycles. Subsequent biochemical analyses for serum urea (UREA), creatinine (CREA), alanine aminotransferase (ALT), aspartate aminotransferase (AST), glucose (GLU), triglycerides (TG), total cholesterol (TC), high-density lipoprotein cholesterol (HDL-C), and low-density lipoprotein cholesterol (LDL-C) were performed using an automatic biochemical analyzer (Mindray, Shenzhen, China; BS-400) in accordance with the manufacturer’s standard protocols. All detection kits were purchased from Nanjing Jiancheng Bioengineering Institute, Nanjing, China.

### Immunocytochemistry, immunohistochemistry, and immunofluorescence

For immunocytochemical staining, cells were seeded onto coverslips in 24-well plates and fixed with 4% PFA for 20 min at room temperature upon reaching 60%–70% confluency. After PBS washing, cells were blocked with 1 × PBS containing 5% normal goat serum and 0.3% Triton X-100 for 1 h at room temperature, then incubated with primary antibody at 4 °C overnight. The primary antibodies included anti-Ac-α-tubulin (Lys40) (#5335T, 1:800, CST, Danvers, MA), anti-α-tubulin (#3873T, 1:1000, CST), anti-lysosomal-associated membrane protein 2 (LAMP2) (#66301-1-Ig, 1:100, Proteintech, Wuhan, China), and anti-CTSB (#31718, 1:400, CST). After PBS washing, secondary antibodies such as Alexa Fluor™ 647 Goat Anti-Rabbit (# A31634, 1:500, Invitrogen) and Alexa Fluor™ 488 Goat Anti-Mouse (#A11029, 1:500, Invitrogen) were added for 1 h at 37 °C in the dark. Coverslips were mounted with DAPI-containing antifade reagent (#BL739B, Biosharp, Hefei, China). Confocal microscopy was used for imaging. Fiji software quantified perinuclear and whole-cell fluorescence intensities of Ac-α-tubulin and LAMP2, and Pearson’s colocalization coefficient of CTSB and LAMP2.

For immunohistochemical staining, paraffin-embedded brain sections were dewaxed in xylene at 60 °C for 1 h and rehydrated with a graded ethanol series (100%–75%). Antigen retrieval was performed by boiling in sodium citrate buffer (#P0081, Beyotime) at 95 °C for 15 min, followed by endogenous peroxidase blocking with 3% H_2_O_2_ at room temperature. Sections were blocked with 1 × PBS containing 5% normal goat serum and 0.3% Triton X-100 at 37 °C for 30 min, then incubated with primary antibody for 1–2 h at 37 °C. The primary antibodies included anti-NDST3 (#NBP2-19501, 1:100, Novus, Littleton, CO), anti-6E10 (#803001, 1:300, Biolegend, San Diego, CA), and anti-p-tau (Ser422) (#GTX86147, 1:100, GeneTex, San Antonio, TX). Staining was completed in accordance with the manufacturer’s instructions for the mouse/rabbit IgG immunohistochemistry kit (#SA1020, Boster Biological Technology, Wuhan, China). Microscopic imaging was performed, and integrated optical density values were quantified using Fiji software and the Immunohistochemistry Profiler plugin. Relative expression was normalized to NonTg or Con group (set to 1.0) as the ratio of mean optical density.

For immunofluorescence, paraffin-embedded brain sections were dewaxed, rehydrated, antigen retrieved and blocked as for immunocytochemical staining. Sections were then incubated with primary antibody at 4 °C for 12–16 h. The primary antibodies included anti-NDST3 (#NBP2-19501, 1:100, Novus), anti-NeuN (#94403, 1:300, CST), anti-Iba1 (#ab283319, 1:100, Abcam, Cambridge, MA), anti-GFAP (#3670, 1:200, CST), anti-MBP (#BF8010, 1:100, Affinity, Cincinnati, OH), anti-CD68 (#HY-P86360, 1:200, MCE, Monmouth Junction, NJ); anti-Ac-α-tubulin (Lys40) (#5335T, 1:800, CST), and anti-Iba1 (#OB-MMS039, 1:500, Oasis Biofarm, Hangzhou, China). After PBS washing, sections were incubated with Alexa Fluor™ 647 Goat Anti-Rabbit (#A31634, 1:500, Invitrogen) or Alexa Fluor™ 488 Goat Anti-Mouse (#A11029, 1:500, Invitrogen) at 37 °C for 30 min in the dark. Afterward, DAPI-containing antifade mounting medium (#BL739B, Biosharp) was applied, and the slides were sealed. Confocal microscopy was used for imaging. Fiji software quantified fluorescence intensity, positive area, Pearson’s colocalization coefficient and colocalized area of target signals.

### Determination of lysosomal pH in vivo

To observe lysosomal pH changes in neurons within the mouse brain, the adeno-associated virus serotype 9 (AAV9) with neuronal specificity, expressing hSyn-mCherry-GFP-LC3B (Obio Technology, Shanghai, China; 5.88 × 10^12^ vg/mL), was stereotactically injected into the lateral ventricle of 9-month-old mice. After sodium pentobarbital (#P3761, Sigma-Aldrich, St. Louis, MO; 80 mg/kg) anesthesia, the mouse was fixed in a stereotactic device. According to coordinates (0.4 mm posterior, ± 1 mm lateral and 3 mm ventral relative to bregma), a hole was drilled in the skull, and 1 µL/side of the virus was microinjected into the lateral ventricle. Brain tissue was collected for observation after 1 month of virus expression as described previously [21]. Briefly, reagents were prepared according to the formulations of Perfusion Wash Super Reagent (#1222SK, Electron Microscopy Sciences, Morgantown, PA) and Perfusion Fixative Super Reagent (#1223SK, Electron Microscopy Sciences). After deep anesthesia, the mice were perfused with Perfusion Wash Super Reagent via the apex of the heart, followed by fixation with Perfusion Fixative Super Reagent. The brain was dissected and further fixed for 24 h in Perfusion Fixative Super Reagent, after which 40-µm thick coronal sections were prepared using a vibratome (Leica, VT1200S). The brain sections were further blocked and immunostained with primary antibody CTSB (#31718, 1:400, CST) for 12–16 h, followed by incubation with Alexa Fluor™ 647 Goat Anti-Rabbit (#A31634, 1:500, Invitrogen) in the dark. After mounting, imaging was performed using confocal microscopy (Nikon, Tokyo, Japan; AXR), with the parameters set as follows: GFP (ex: 488 nm, em: 490–560 nm), mCherry (ex: 561 nm, em: 600–650 nm), Alexa Fluor 647 (ex: 633 nm, em: 640–710 nm). The CA1 subregion of the caudal hippocampus was selected as the region of interest (ROI) for analysis. Images were acquired using a 63 × oil objective. Poorly acidified lysosomes appeared yellow after hSyn-mCherry-GFP-LC3B expression, and when combined with the CTSB signal (pseudo-colored as blue), they appeared white. Fiji software was used to count the number of white puncta. Data were collected from two non-consecutive sections  (80 µm interval) per mouse (three mice per group), with five random fields quantified per section.

### Western blotting assay

Mouse hippocampus tissues or cells were fully lysed in RIPA lysis buffer (#P0013B, Beyotime) containing 1 mM PMSF (#ST506, Beyotime) and 1 × protease inhibitor (#P1005, Beyotime). Lysates were centrifuged at 12,000 rpm for 20 min at 4 °C, and the supernatants were collected for subsequent assays. The protein concentrations were adjusted to the same level across groups based on the BCA assay results. Equal amounts of protein (30 µg) were separated on a 10% or 15% Tris–glycine SDS-PAGE gel and transferred onto nitrocellulose blotting membranes, with the exception of Aβ detection, which was performed using 16% Tricine SDS-PAGE (loading volume adjusted to 60 µg as well). The membranes were blocked with 5% skim milk at room temperature for 2 h and incubated with indicated primary antibody. The primary antibodies used in this study were as follows: ATP6V1A (#sc-293336, 1:500, Santa Cruz Biotechnology, Dallas, TX), ATP6V1C1 (#sc-271077, 1:500, Santa Cruz Biotechnology), ATP6V0D (#sc-393322, 1:500, Santa Cruz Biotechnology), LAMP2 (#BS61513, 1:1000, Bioworld, Bloomington, MN), 6E10 (#803015, 1:1000, Biolegend), APP (#ab32136, 1:1000, Abcam), APP-C-Terminal (#A8717, 1:1000, Sigma-Aldrich), GAPDH (#AF7021, 1:5000, Affinity), α-tubulin (#11224-1-AP, 1:5000, Proteintech), MAPT/tau (#ab80579, 1:1000, Abcam), p-MAPT/tau (Ser262) (#AF3151, 1:1000, Affinity), p-MAPT/tau (Thr212) (#AF3146, 1:1000, Affinity), p-MAPT/tau(Ser422) (#GTX86147, 1:1000, GeneTex), β-actin (#AF7018, 1:5000, Affinity), HDAC6 (#AF6485, 1:1000, Affinity), LC3B (#ab192890, 1:2000, Abcam), SQSTM1/P62 (#5114, 1:1000, CST), GM130 (#HY-P82075, 1:1000, MCE), Calnexin (#HY-P80578, 1:1000, MCE), VDAC1 (#HY-P80369, 1:10000, MCE), and NDST3 (#NBP2-19501, 1:1000, Novus) at 4 °C for 12–16 h. After washing with PBS, the membrane was incubated with HRP-conjugated or IRDye-conjugated secondary antibody at room temperature for 1 h. The secondary antibodies used in this study were as follows: HRP-conjugated Goat Anti-Mouse IgG (#SA00001-1, 1:5000, Proteintech), HRP-conjugated Goat Anti-Rabbit IgG (#SA00001-2; 1:5000, Proteintech), IRDye 680LT donkey anti-mouse IgG secondary antibody (#926-68022, 1:10000, LI-COR, Lincoln, NE), and IRDye 800CW goat anti-rabbit IgG secondary antibody (#926–32211, 1:10000, LI-COR). Immunoblots were analyzed for band intensity using either ECL chemiluminescence substrate (#4AW011-500, Beijing 4A Biotech, Beijing, China) with gel imaging system (CLINX, Shanghai, China; ChemiScope 6200 Touch) or a LI-COR Odyssey CLx Infrared Imaging System (LI-COR).

### Thioflavine S staining

Paraffin-embedded mouse brain tissue sections were first dewaxed and rehydrated as described in the immunohistochemistry protocol. The sections were then incubated in 0.1% thioflavine-S solution (#T1892, Sigma-Aldrich) in the dark for 15 min to stain amyloid plaques. After incubation, the sections were washed twice in 80% alcohol, followed by a single wash in distilled water. The slides were mounted, dried in the dark to prevent photo-bleaching, and then imaged using a fluorescence microscope (Leica, DMI8 THUNDER). Amyloid plaques were quantitatively analyzed using the Fiji software.

### Fluoro-Jade C (FJC) staining

Degenerating neurons were detected using the FJC staining kit (#TR-100-FJT, Biosensis, Thebarton, SA, Australia), following the manufacturer’s instructions with minor modifications. Briefly, paraffin-embedded mouse brain tissue sections were dewaxed, rehydrated, and subjected to antigen retrieval following the standard immunohistochemistry protocol. After rinsing in distilled water for 2 min, sections were incubated in reagent B (diluted 1:20) for 5 min. Subsequent to washing with distilled water, sections were incubated with reagent C (diluted 1:10) at room temperature for 10 min in the dark, followed by three rinses in distilled water. The sections were then incubated in a freshly prepared crosslinking buffer containing 10 mM 1-(3-Dimethylaminopropyl)-3-ethylcarbodiimide (EDC; #D836336, Macklin, Shanghai, China) and 25 mM N-Hydroxysuccinimide (NHS; #H6231, Macklin) at room temperature for 10 min. After washing sequentially with MES buffer and distilled water, sections were processed for NeuN immunofluorescence staining as described above. Finally, sections were counterstained with DAPI and mounted using neutral anti-fade mounting medium. Images were acquired using a fluorescence microscope.

### Transmission electron microscopy (TEM)

For mouse hippocampal sections, mice were deeply anesthetized and transcardially perfused with 2% glutaraldehyde (#G6257, Sigma-Aldrich). The intact hippocampus was dissected from the whole brain, cut into 1 mm^3^ cubes, and immediately fixed with 2% glutaraldehyde for 24 h. After PBS washing, the tissue was fixed with 1% osmium tetroxide (OsO4) (#251755, Sigma-Aldrich) at room temperature for 2 h. The tissue blocks were dehydrated in graded alcohol and embedded in epoxy resin (#31185, Sigma-Aldrich), then sectioned into 100-nm ultrathin slices using an ultramicrotome. Staining was performed with uranyl acetate (#YS25690U, Shanghai Yaji Biological Technology, Shanghai, China) and lead citrate (#L885990, Macklin), and imaging was done via a transmission electron microscope (Hitachi, Tokyo, Japan; H-7500).

For purified lysosomes, the lysosome suspension was diluted to an appropriate concentration, and a 5 µL aliquot was dropped onto a carbon-coated copper grid. After incubation for 2 min to allow full adsorption, excess liquid was blotted off gently with filter paper. Subsequently, the grid was incubated with 2% phosphotungstic acid (PTA, pH 6.8) for negative staining. After air-drying, lysosome morphology was observed via a transmission electron microscope (JEOL, Tokyo, Japan; Jem-1400plus).

### Golgi staining

According to the manufacturer’s instructions for the FD Rapid GolgiStain™ Kit (#PK401, FD Neuro Technologies, Columbia, MD), mice were euthanized via an overdose of inhaled isoflurane followed by cervical dislocation, and the whole brain was dissected into left and right halves along the longitudinal fissure, then soaked in a pre-prepared mixture of solution A and solution B for 2 weeks. Subsequently, the brain was then soaked in solution C for 3–5 days. The brain was carefully placed in − 80 °C isopentane for rapid freezing. The frozen tissue was sectioned coronally into 100 μm-thick slices using a cryostat microtome, mounted onto gelatin-coated slides treated with 10% gelatin and 0.05% chromium potassium sulfate, and dried in the dark at room temperature for 3 days. The sections were stained using the staining solution from the kit, cleared in xylene three times, and mounted with neutral balsam. The density of dendritic spines was analyzed using Fiji software.

### Nissl staining

Paraffin-embedded mouse brain tissue sections were dewaxed and rehydrated as described in the immunohistochemistry protocol. The sections were then incubated in Nissl staining solution (#C0117, Beyotime) at room temperature for 15 min. Following incubation, the sections were washed with distilled water, dehydrated through a graded alcohol series (70%–100%), and treated with xylene twice for clearing. Finally, the sections were mounted with neutral balsam. Images were captured using an optical microscope, and Fiji software was used to count the number of normal neurons.

### Morris water maze test

The Morris water maze was used to assess the spatial learning and memory ability of 10-month-old mice, as described previously [34, 35]. A circular pool with a diameter of 120 cm and a depth of 50 cm was filled with tap water containing food-grade titanium dioxide, and the water temperature was maintained at 21 ± 2 °C. The pool was divided into four quadrants, and labels with different colors and shapes were placed on the walls of each quadrant. On the day before the experiment, each mouse was given a 60-s free-swimming session to adapt to the maze. During days 1–5 (spatial learning phase), the platform was set 1.5 cm below the water surface, and the mice were trained to find the platform four times a day. If the mice did not find the platform within 60 s, they were guided by the experimenter to the platform to stay for 15 s. On the 6th day (probe phase), the platform was removed, and the mice were allowed to swim freely for 60 s. All experimental data were recorded using the SMART v3.0 tracking system (RWD Life Science Co., Ltd).

### Novel object recognition test

The novel object recognition test was used to evaluate the recognition memory ability of mice [36, 37]. The day before the formal experiment, each mouse was allowed to freely explore a 50 cm × 50 cm × 50 cm plastic apparatus for 5 min to adapt to the environment. Twenty-four hours later (familiarization phase), two objects, O1 and O2, identical in shape and color and 7 cm in diameter, were placed 5 cm from the inner wall of the plastic apparatus. Each mouse was placed inside and allowed to freely explore for 5 min. Six hours later (test phase), one of the old objects was retained as O3, while the other was replaced with a new object, N, of the same size but a different shape and color. Each mouse was again placed in the apparatus for free exploration for 5 min. To eliminate the influence of the animal odor on the experiment, after each test, the feces and urine were removed, and the plastic apparatus was cleaned with 75% alcohol. Mouse recognition was defined as the mouse touching the object or being within 2 cm of it, and the exploration time of each object was recorded using the SMART v3.0 tracking system (RWD Life Science Co., Ltd). The ratio of the difference in exploration time between the new object and the old object (N-O3) to the total exploration time (N + O3) was defined as the discrimination index.

### Quantification and statistical analyses

Unless otherwise specified, images for histological and immunofluorescence quantification were acquired from four non-consecutive sections per mouse at 80-µm intervals throughout the caudal hippocampus to ensure unbiased sampling. The CA1 and DG subregions were defined as ROIs based on anatomical landmarks [38]. For large-scale and distinct signals (e.g., plaques, GFAP, Iba1), the CA1 and DG signals were combined for quantification. For small-scale, faint signals or those requiring colocalization analysis (e.g., Ac-α-tubulin, CD68/Iba1, FJC/NeuN), images were randomly acquired using a high-magnification objective, and signals in the CA1 and DG regions were analyzed separately. All data are presented as mean ± standard error of the mean (SEM). The data were analyzed and plotted using GraphPad Prism 9 software. Data from two groups were analyzed using an unpaired two-tailed Student’s *t*-test, and data from three or more groups were analyzed by one-way or two-way analysis of variance (ANOVA) followed by Dunnett’s or Tukey’s post-hoc tests. The exact analysis methods are indicated in the corresponding figure legends. All statistical analyses were pretested for normality. A *P* value < 0.05 was considered statistically significant.

## Results

### NDST3 regulates lysosomal pH comparable to HDAC6, despite a distinct subcellular spatial pattern in catalyzing microtubule deacetylation

Although both having the function of microtubule deacetylation, NDST3 and HDAC6 exhibited distinct catalytic patterns. Immunostaining for acetylated α-tubulin (Ac-α-tubulin) revealed that hyperacetylated microtubules in *HDAC6* KO RPE1 cells were uniformly distributed along the microtubule network, whereas *NDST3* KO RPE1 cells showed pronounced perinuclear accumulation of hyperacetylated microtubules (Fig. S1a–f). As RPE1 is a retinal pigment epithelial cell line, it remained unclear whether the observed phenomenon is conserved in brain cells.

**Fig. 1:**
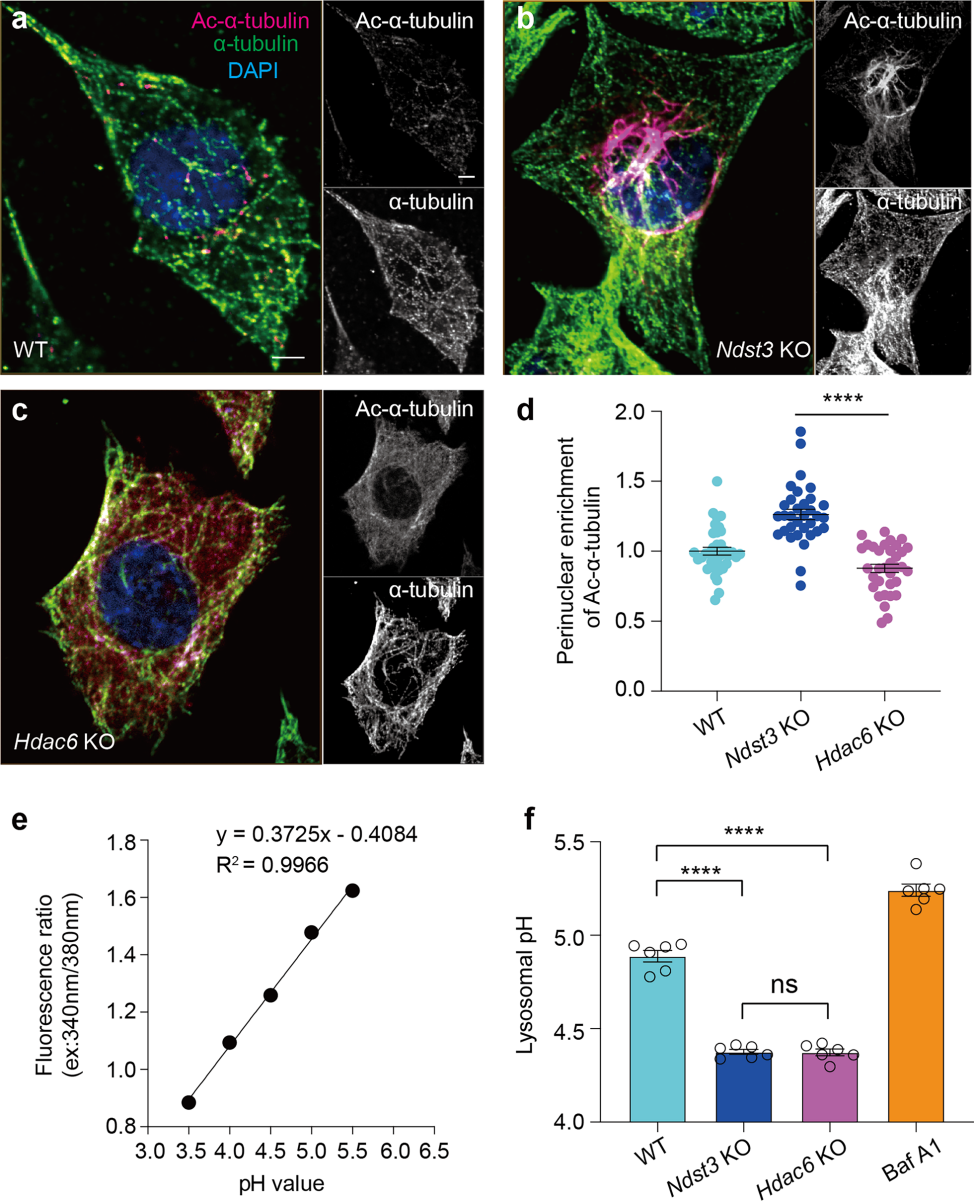
NDST3 and HDAC6 induce spatially distinct microtubule deacetylation, yet exerting similar regulation of lysosomal pH in HT22 cells. **a**–**c** Representative immunofluorescence staining images of acetylated-α-tubulin (Ac-α-tubulin) and total α-tubulin in wild-type (WT) (**a**), *Ndst3* KO (**b**) and *Hdac6* KO (**c**) cells. Scale bars, 5 μm (main panels and insets). **d** Perinuclear enrichment of Ac-α-tubulin (5-μm perinuclear region, intensity ratio) calculated from **a**–**c** (*n* = 36 cells, WT; *n* = 33 cells, *Ndst3* KO; *n* = 34 cells, *Hdac6* KO; ****P < 0.0001). **e** Calibration curve for lysosomal pH measurement in HT22 cells (*n* = 6 independent cultures). **f** Lysosomal pH values calculated from the fluorescence ratio measured by LysoSensor™ yellow/blue DND-160 staining on the basis of the pH calibration curve in **e** (*n* = 6 independent cultures; ****P < 0.0001, ns represents nonsignificant). Bafilomycin A1 (Baf A1; 100 nM, 10 min) served as a positive control. Error bars represent SEMs. Two-tailed Student’s *t* tests (**d**), or one-way ANOVA followed by Tukey's post hoc test (**f**)

In the mouse brain, NDST3 displayed neuron-specific expression, with minimal detection in glial cells (Fig. S1g, h). This neuron-restricted expression pattern was corroborated by independent single-cell transcriptomics datasets (http://www.alzdata.org/) [39]. Therefore, we further compared the functional differences between NDST3 and HDAC6 in a mouse hippocampal neuronal cell line HT22. We generated *Ndst3* KO and *Hdac6* KO HT22 cells (Fig. S1i) and performed immunofluorescence staining against Ac-α-tubulin. Consistent with our observations in RPE1 cells, *Ndst3* KO and *Hdac6* KO HT22 cells exhibited distinct distributions of hyperacetylated microtubules. Specifically, depletion of NDST3 resulted in significantly greater perinuclear enrichment of Ac-α-tubulin compared to HDAC6 ablation (Fig. 1a–d). Notably, this NDST3-mediated perinuclear microtubule deacetylation aligned with its characteristic perinuclear subcellular localization (Fig. S1j), providing a spatial basis for its specific regulation of microtubule acetylation in the perinuclear region.

Although NDST3 and HDAC6 exhibited distinct catalytic patterns in microtubule deacetylation, their depletion resulted in comparable lysosomal pH levels. We used a ratiometric probe, LysoSensor Yellow/Blue DND-160, to examine lysosomal pH in WT, *Ndst3* KO and *Hdac6* KO HT22 cells. This probe has pH-dependent dual excitation properties, enabling reliable measurement of the exact lysosomal pH values when paired with a pH calibration curve [20, 23, 27]. LysoSensor measurements revealed that the lysosomal pH values in both *Ndst3* KO and *Hdac6* KO HT22 cells were significantly lower than those in their respective WT controls. However, there was no significant difference in lysosomal pH between the two KO cell lines (Fig. 1e, f). Similar results were also observed in *NDST3* KO and *HDAC6* KO RPE1 cells (Fig. S1k, l). These findings align with our previous report demonstrating that lysosomal acidification is highly dependent on perinuclear microtubule acetylation [27], further validating the functional link between microtubule acetylation status and lysosomal acidification.

### NDST3 is a regulator of lysosomal acidification in HT22 cells with APP695^Swe^ overexpression

**Fig. 2:**
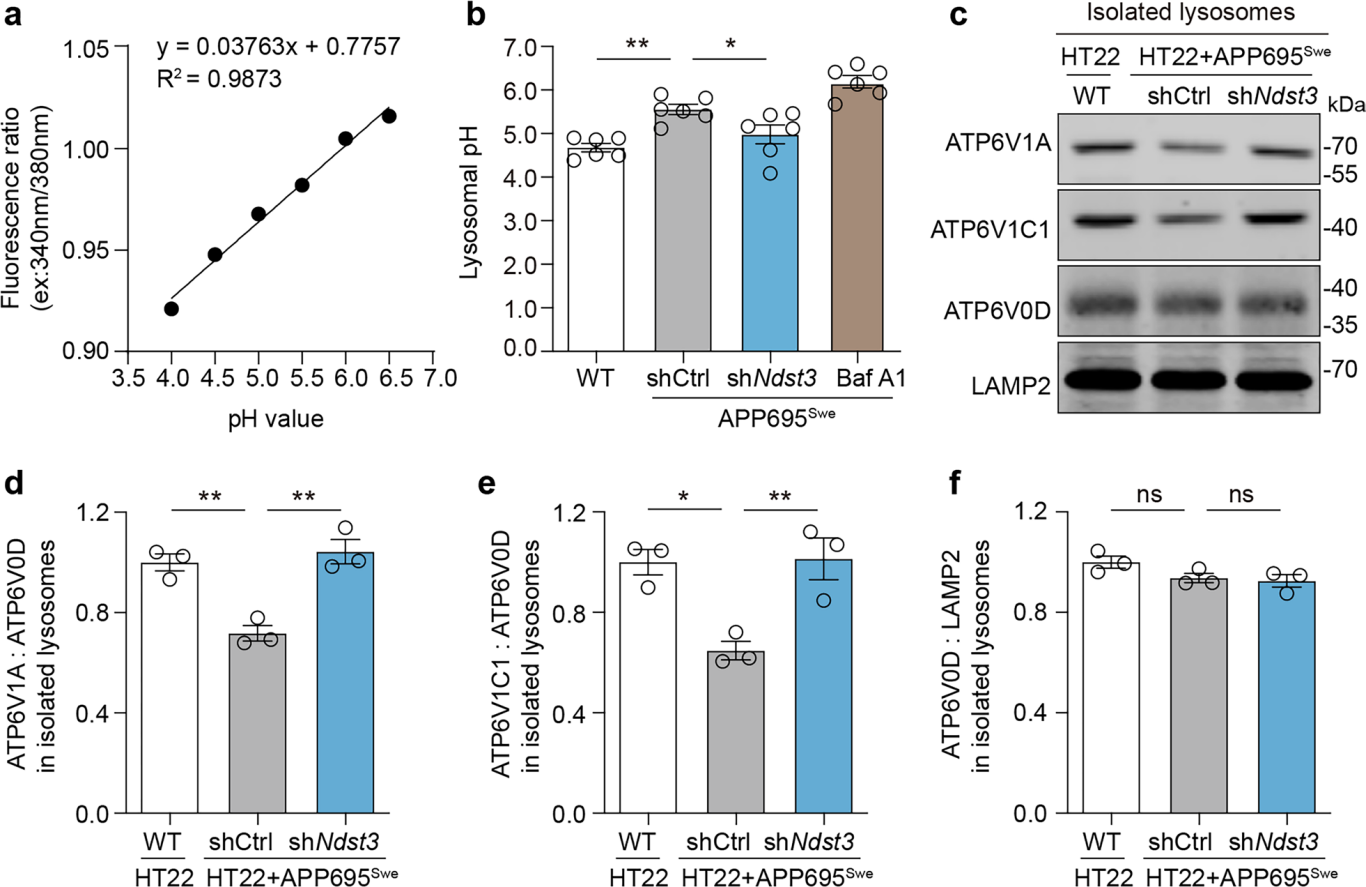
NDST3 deficiency promotes lysosomal acidification in HT22 cells with APP695^Swe^ overexpression. **a** Calibration curve for lysosomal pH measurement in HT22 cells (*n* = 6 independent cultures). **b** Lysosomal pH calculated from the fluorescence ratio of LysoSensor™ yellow/blue DND-160 staining, based on the pH calibration curve in **a** (*n* = 6 independent cultures; *P = 0.0326, **P = 0.0020). **c** Immunoblots of V-ATPase subunits (ATP6V1A, ATP6V1C1, and ATP6V0D) in isolated lysosomes from control HT22 cells and APP695^Swe^-overexpressing HT22 cells transfected with shRNA against *Ndst3* (sh*Ndst3*) or scramble shRNA (shCtrl). LAMP2 was used as a loading control. **d**, **e** Ratios of ATP6V1A (**d**) or ATP6V1C1 (**e**) to ATP6V0D in lysosomal fractions, reflecting V-ATPase V1 domain docked to the V0 domain (*n* = 3 independent experiments; *P = 0.0110, **P < 0.01). **f** Quantitative analysis of ATP6V0D levels relative to LAMP2 levels in lysosomal fractions (*n* = 3 independent experiments, ns represents nonsignificant). Error bars represent the SEMs. One-way ANOVA followed by Dunnett’s post hoc tests

While HDAC6 has been rigorously pursued as a therapeutic target for AD [40], the role of NDST3 in this disease remains unexplored. To investigate the potential involvement of NDST3 in AD pathogenesis, we established an in vitro cell model by transducing a construct expressing a mutant form of APP, APP695^Swe^, into HT22 cells. The HT22 cells with APP695^Swe^ overexpression exhibited typical features of AD pathogenesis, including altered APP processing characterized by APP-CTFβ accumulation (Fig. S2a, b) and increased vulnerability to cell death (Fig. S2c, d). Subsequently, we used an shRNA against *Ndst3* mRNA (hereafter referred to as sh*Ndst3*) to knock down NDST3 expression in the APP695^Swe^ model. A scrambled shRNA was used as a negative control (shCtrl). The knockdown efficiency of *Ndst3* was validated by western blot analysis (Fig. S2e, f). We subsequently examined the lysosomal pH in the cells. WT HT22 cells were used as normal controls. As indicated by the LysoSensor probe, the lysosomal pH of the HT22 cells was approximately 4.7 under physiological conditions, whereas the lysosomal pH of the APP695^Swe^ model cells significantly increased to 5.6. Treatment with sh*Ndst3* reacidified the APP695^Swe^ cells, lowering the lysosomal pH to less than 5.0. (Fig. 2a, b).

Next, we investigated whether NDST3 knockdown affects the assembly of the lysosomal V-ATPase V1–V0 holoenzyme in the APP695^Swe^-overexpressing HT22 cell model. To this end, we isolated lysosomal fractions from WT, shCtrl-, and sh*Ndst3*-treated APP695^Swe^ cells. The purity of the isolated lysosomes was validated by immunoblotting for the lysosomal marker LAMP2, with near-absence of contamination from other organelles, including the Golgi apparatus (GM130), endoplasmic reticulum (Calnexin), mitochondria (VDAC1), and cytoplasm (GAPDH) (Fig. S2g). The success of lysosome isolation was further corroborated by TEM analysis and LysoTracker staining (Fig. S2h, i). Then, we analyzed the levels of V1 and V0 subunits in the isolated lysosomal fractions. The holoenzyme level was determined as the levels of the lysosome-bound V1 subunits V1A (ATP6V1A) and V1C1 (ATP6V1C1) relative to the level of the V0 subunit V0D (ATP6V0D). Compared with WT cells, the levels of ATP6V1A and ATP6V1C1 in the lysosomal fraction of APP695^Swe^-overexpressing cells were significantly lower; however, this decrease was reversed by treatment with sh*Ndst3* (Fig. 2c–e). The level of ATP6V0D on lysosomes did not significantly differ across the three groups (Fig. 2c, f). The total levels of ATP6V1A, ATP6V1C1, and ATP6V0D in whole-cell lysates were unaffected by the treatments (Fig. S2j–m). These results suggest that NDST3 deficiency promotes the assembly of the V-ATPase V1–V0 holoenzyme, providing additional evidence for the regulation of lysosomal acidification by NDST3 in HT22 cells with APP695^Swe^ overexpression.

### Restoration of lysosomal acidity via NDST3 knockdown enhances the autophagic degradation of aberrant Aβ and MAPT/tau in HT22 cells with APP695^Swe^ or MAPT/tau^P301L^ overexpression

**Fig. 3:**
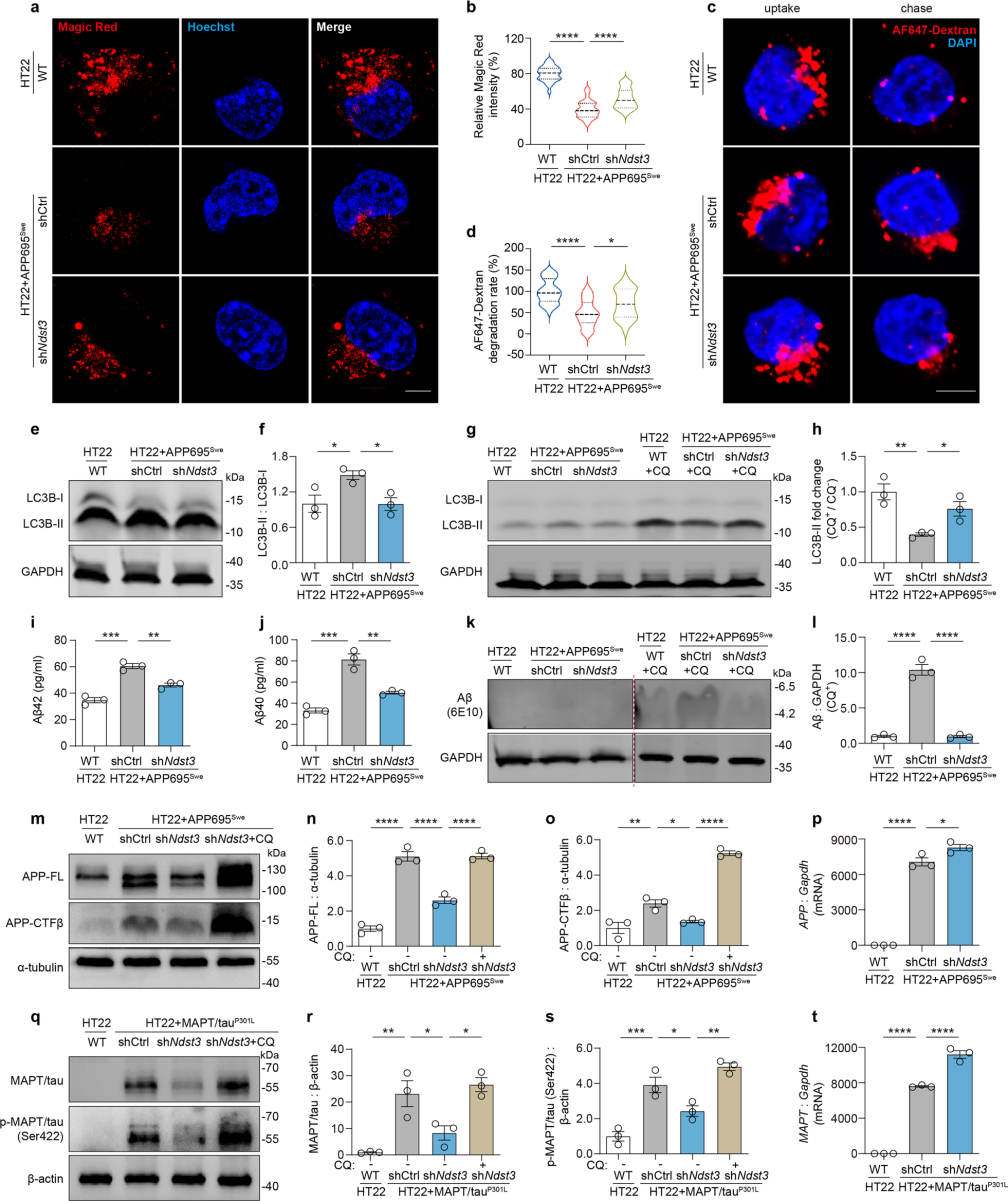
Silencing *Ndst3* rescues lysosomal function and degrades aberrant Aβ and MAPT/tau in HT22 cells. **a**, **b** Magic Red assay for CTSB activity. The Magic Red CTSB substrate, MR-(RR)2, generates red fluorescence when excited at 550 nm following enzymatic cleavage by active CTSB. Representative images of Magic Red staining are shown in **a**. Scale bar, 5 μm. The relative Magic Red intensity per cell was quantified (**b**) (*n* = 30 cells in each group; ****P < 0.0001). **c**, **d** Assessment of lysosomal degradative capacity using Alexa Fluor 647 (AF647)-dextran. The AF647 fluorescence was measured at dextran endocytosis (uptake) and 4-h post-endocytosis (chase), with degradation rate calculated as indicated in *Materials and methods*. Scale bar, 5 μm. *n* = 30 cells per group; *P = 0.0151, ****P < 0.0001. **e**, **f** LC3 conversion assessed by immunobloting of LC3B-II/I in HT22 WT, shCtrl-, and sh*NDST3*-treated APP695^Swe^ cells (*n* = 3 independent experiments; *P < 0.05). **g**, **h** LC3 turnover assay. Degradation of LC3B-II was estimated by comparing samples with and without chloroquine (CQ; 50 μM, 4 h) treatment. LC3B-II level changes were quantified to assess the autophagic flux (*n* = 3 independent experiments; *P = 0.0499, **P = 0.0056). **i**, **j** Measurement of extracellular Aβ42 (**i**) and Aβ40 (**j**) levels in cell culture media via ELISA assay (*n* = 3 independent cultures; **P < 0.01, ***P < 0.001). **k**, **l** Immunoblot analysis of intracellular Aβ (6E10 antibody) in HT22 WT, shCtrl-, and sh*NDST3*-treated APP695^Swe^ cells with or without CQ treatment (*n* = 3 independent experiments; ****P < 0.0001). **m–o** Immunoblot analysis of full-length APP (APP-FL) and APP-CTFβ (*n* = 3 independent experiments; *P = 0.0295, **P = 0.0059, ****P < 0.0001). CQ refers to chloroquine (50 μM, 4 h), which was used to block the autophagy-lysosomal pathway. **p** qPCR assay for *APP* mRNA levels in HT22 WT, shCtrl-, and sh*NDST3*-treated APP695^Swe^ cells (*n* = 3 independent experiments; *P = 0.0256, ****P < 0.0001). **q–s** Immunoblot analysis of MAPT/tau and p-MAPT/tau (Ser422) (*n* = 3 independent experiments; *P < 0.05, **P < 0.01, ***P < 0.001). β-Actin was used as a loading control. **t** qPCR assay for *MAPT* mRNA levels in HT22 WT, shCtrl-, and sh*NDST3*-treated MAPT/tau^P301L^ cells (*n* = 3 independent experiments; ****P < 0.0001). GAPDH, α-tubulin, and β-actin served as the loading control in immunoblot analysis. *Gapdh* mRNA served as the internal controls in qPCR assays. Error bars represent the SEMs. One-way ANOVA with Dunnett’s post hoc tests, or Tukey’s post hoc tests (for **n**, **o**, **r**, and **s**)

LAMP2 immunofluorescence staining demonstrated a reduction of lysosomes in the perinuclear region (where autophagosomes accumulate and most autophagosome-lysosome fusion occurs [41, 42]), in APP695^Swe^-transduced HT22 cells compared to WT controls. Notably, this deficit was substantially rescued by NDST3 knockdown (Fig. S3a, b). It is widely recognized that the perinuclear lysosomes are more acidic, which enhances enzymatic function and autophagic degradation [41]. Hence, we further investigated whether NDST3 regulates lysosomal degradation capacity, thereby affecting the clearance of toxic misfolded proteins (Aβ and MAPT/tau) in HT22 cells expressing APP695^Swe^ or MAPT/tau^P301L^. CTSB is a typical lysosomal protease that is responsible for the degradation of various substrates, including AD-associated proteins [10]. We measured CTSB activity using the Magic Red CTSB Kit as described in our previous study [27]. Compared with WT cells, APP695^Swe^-overexpressing cells subjected to null treatment presented a markedly decreased Magic Red fluorescence signal (red). However, after undergoing sh*Ndst3* treatment, the APP695^Swe^-overexpressing cells presented a significant increase in the Magic Red signal (Fig. 3a, b). The total level of the lysosomal marker LAMP2 remained unchanged after sh*Ndst3* treatment (Fig. S3a, c). Additionally, the amount of CTSB that colocalized with LAMP2 was comparable between the treated and the untreated cells (Fig. S3a, d). These findings suggest that the observed increase in CTSB activity resulted from neither an increase in the number of lysosomes nor an increase in CTSB levels within lysosomes, but instead from the restoration of lysosomal acidity.

To further assess the degradative capacity of lysosomes, we utilized a pH-insensitive AF647-dextran probe, which is internalized by cells via endocytosis and subsequently degraded within lysosomes. Fluorescent dextran uptake was comparable across WT cells, APP695^Swe^-overexpressing cells, and sh*Ndst3*-treated APP695^Swe^ cells. Following a 4-h chase period, the fluorescence signal persisted in APP695^Swe^ model cells, but was attenuated in WT cells and sh*Ndst3*-treated APP695^Swe^ cells (Fig. 3c). Quantitative analysis confirmed a significant defect in dextran degradation in APP695^Swe^ cells compared to WT control cells, which was rescued by sh*Ndst3* treatment (Fig. 3d).

The conversion of LC3B-I to LC3B-II was increased in APP695^Swe^-overexpressing cells when compared to WT HT22 cells, indicating that the autophagy-lysosomal pathway was either induced or suppressed at the late stage (Fig. 3e, f). Notably, the LC3B-II/LC3B-I ratio was significantly lower in the sh*Ndst3*-treated APP695^Swe^ cells compared to the model cells. To further clarify the status of autophagic flux, we treated cells in all three groups with chloroquine (CQ), a lysosomal inhibitor that prevents autophagosome degradation, and performed an LC3B-II turnover assay. We observed that the CQ-induced accumulation of LC3B-II was significantly lower in APP695^Swe^ cells than in WT controls; however, sh*Ndst3* treatment restored this accumulation capacity (Fig. 3g, h). Collectively, these data demonstrate that APP695^Swe^ expression compromises autophagy-lysosomal pathway, and this impairment was rescued by NDST3 knockdown.

Next, we investigated whether the rescue of lysosomal function translates to enhanced autophagic degradation of Aβ and MAPT/tau. ELISA analysis demonstrated a significant elevation in extracellular Aβ40 and Aβ42 levels in APP695^Swe^ model cells relative to WT controls; this increase was substantially reversed by sh*Ndst3* treatment (Fig. 3i, j). While intracellular Aβ was undetectable by western blot using the 6E10 antibody under basal conditions, treatment with CQ (100 µM, 6h) induced detectable accumulation (Fig. 3k). Quantification revealed that the CQ-induced Aβ accumulation was significantly higher in APP695^Swe^ cells than in WT controls and sh*Ndst3*-treated cells (Fig. 3l). In addition to Aβ itself, full-length APP (APP-FL) and APP-CTFβ (direct precursors to Aβ) are processed by the lysosomal system [9, 16, 43]. Therefore, we also examined the levels of APP-FL and APP-CTFβ. We observed that the levels of APP-FL and APP-CTFβ in APP695^Swe^-overexpressing HT22 cells were significantly higher than those in WT cells, while these increases were markedly attenuated by sh*Ndst3* treatment (Fig. 3m–o). Intriguingly, CQ treatment abolished the sh*Ndst3*-induced degradation of APP-FL and APP-CTFβ (Fig. 3m–o). qPCR analysis confirmed that the enhanced *APP* mRNA level in APP695^Swe^-overexpressing cells was not reduced by sh*Ndst3* treatment but instead underwent some compensatory upregulation (Fig. 3p). Together, these results suggest a connection between NDST3 knockdown and the autophagic degradation of Aβ and Aβ-related precursors.

Mirroring the effects on Aβ, NDST3 knockdown promoted the autophagic degradation of aberrant MAPT/tau. The levels of total MAPT/tau and phosphorylated MAPT/tau (p-MAPT/tau) at Ser422 (Fig. 3q–s), as well as at Ser262 and Thr212 (Fig. S4a–c), were significantly elevated in MAPT/tau^P301L^-overexpressing HT22 cells compared to WT controls. However, sh*Ndst3* treatment markedly attenuated the increase in MAPT/tau and p-MAPT/tau levels in the model cells. Notably, this inhibitory effect was also abolished by CQ treatment (Fig. 3q–s; Fig. S4a–c). The elevated mRNA level of *MAPT* in MAPT/tau^P301L^-overexpressing cells was also not reversed by sh*Ndst3* treatment (Fig. 3t).

### NDST3 expression is elevated in AD

**Fig. 4:**
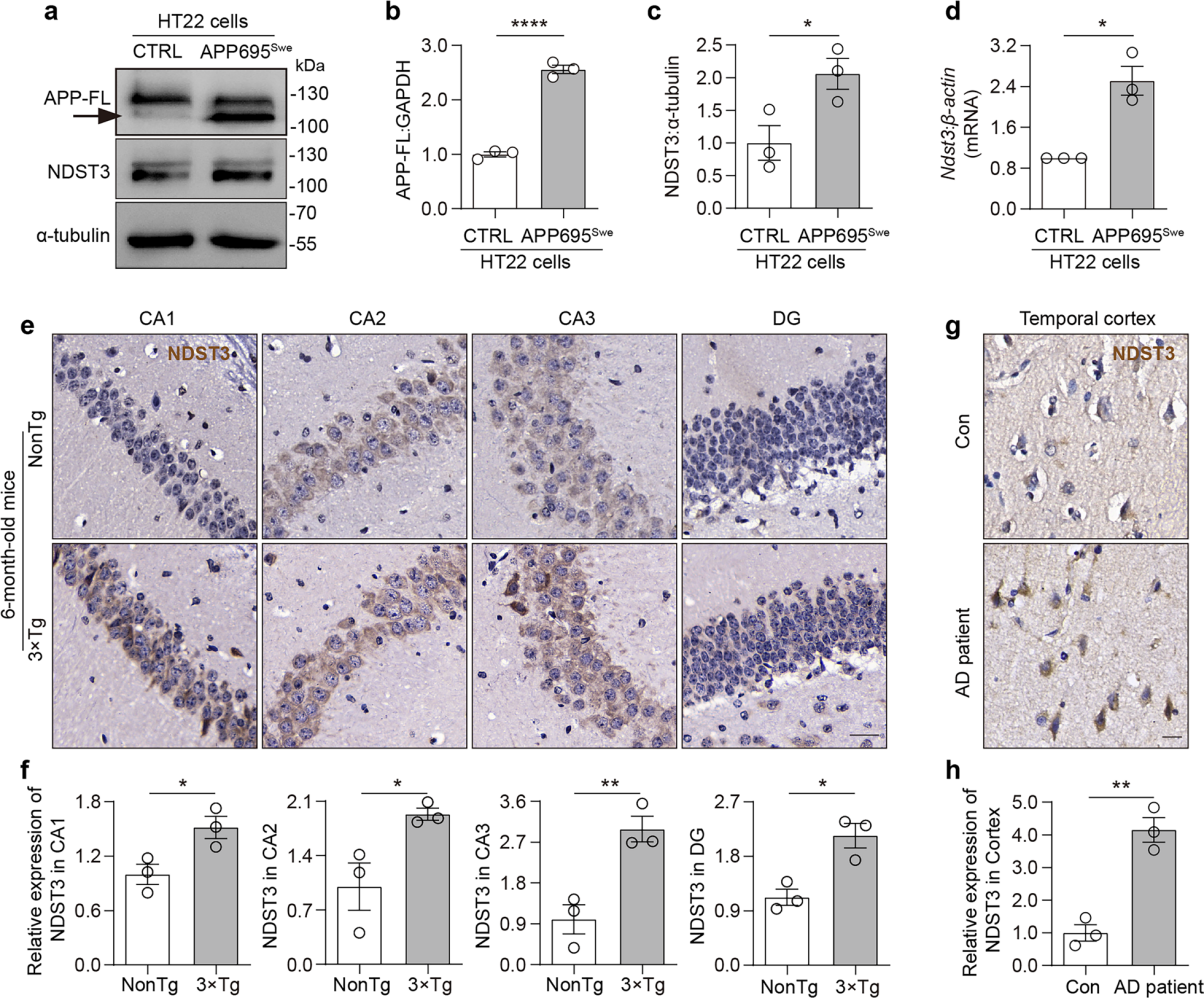
NDST3 is increased in APP695^Swe^-overexpressing HT22 cells, 6-month-old 3 × Tg mice, and AD patients. **a–c** Immunoblot analysis of full-length APP (APP-FL) and NDST3 expression in APP695^Swe^-overexpressing HT22 cells and control (CTRL) cells. α-Tubulin was used as a loading control (*n* = 3 independent experiments; *P = 0.0404, ****P < 0.0001). **d** qPCR assay for *Ndst3* mRNA expression in APP695^Swe^-overexpressing HT22 cells relative to CTRL cells. *β-Actin* served as the internal control (*n* = 3 independent cultures; *P = 0.0058). **e, f** Immunohistochemical staining and quantification of NDST3 expression in the CA1, CA2, CA3, and DG regions of the hippocampus in 6-month-old 3 × Tg-AD and non-transgenic (NonTg) mice. Scale bar, 25 μm. *n* = 3 mice per group; *P < 0.05, **P = 0.0097. **g, h** Immunohistochemical staining and quantification of NDST3 expression in postmortem human brain samples from AD patients and healthy controls (Con). Scale bar, 50 μm. *n* = 3 human subjects per group; **P = 0.0022. Error bars represent SEMs. Two-tailed Student’s t tests

Intriguingly, we detected a pronounced increase in NDST3 expression at both mRNA and protein levels in APP695^Swe^-overexpressing HT22 cells compared to WT cells (Fig. 4a–d). To validate this increase of NDST3 expression in vivo, we examined NDST3 expression in the hippocampus of 3 × Tg-AD mice, a well-established model of AD, using nontransgenic (NonTg) mice as a control. Across different ages (3, 6, and 12 months), NDST3 protein levels were significantly elevated in the hippocampal CA1, CA2, CA3 and DG regions of 3 × Tg-AD mice relative to controls (Fig. 4e, f; Fig. S5a–d). To extend these observations to humans, we analyzed postmortem human brain samples from both AD patients and healthy controls (Table S2). These AD brain samples exhibited characteristic pathological features, such as Aβ deposition (Fig. S5e, f). Importantly, histochemical staining confirmed a significant increase in NDST3 level in AD patients compared with healthy controls (Fig. 4g, h).

Functionally, we found that overexpression of NDST3 (*Ndst3* OE) in HT22 cells led to a significant increase in lysosomal pH compared to the control group (Fig. S5g–i), confirming that NDST3 elevation disrupts lysosomal homeostasis.

### Decreased NDST3 expression restores lysosomal function and alleviates the Aβ and MAPT/tau burdens in 3 × Tg-AD mice

**Fig. 5:**
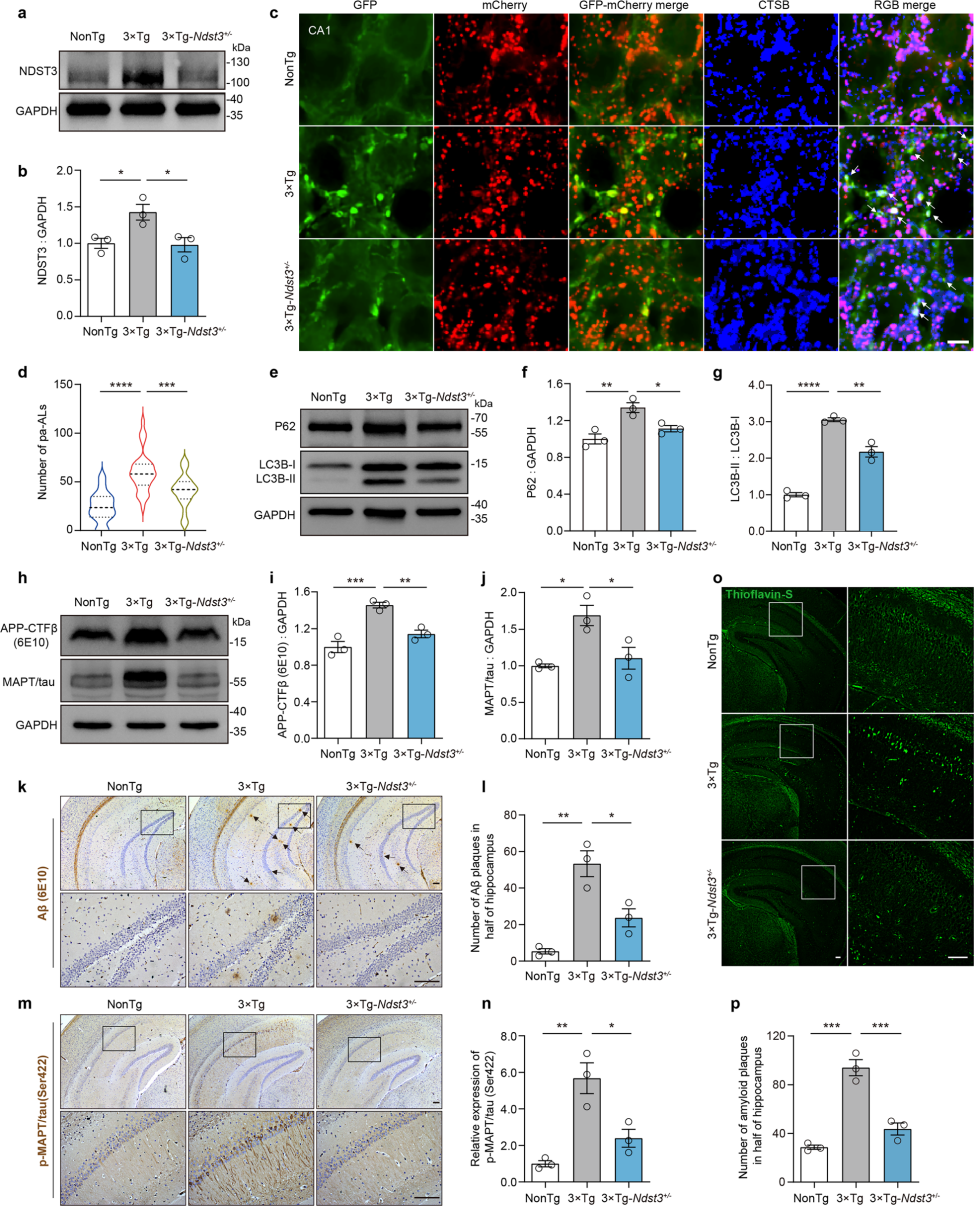
Reduced NDST3 expression restores lysosomal function and alleviates the Aβ and MAPT/tau burdens in 10-month-old 3 × Tg-AD mice. **a**, **b** Immunoblot analysis of NDST3 in the hippocampi of NonTg, 3 × Tg, and 3 × Tg-*Ndst3*^+/−^ mice (*n* = 3 independent experiments; *P < 0.05). **c** Representative fluorescence images of tandem fluorescence-tagged LC3 co-labeled with lysosomal marker CTSB in the CA1 region of mouse hippocampus. Fully acidified autolysosomes (f-ALs) appear purple, whereas poorly acidified autolysosomes (pa-ALs, indicated by the white arrows) appear white. Scale bar, 5 μm. **d** Quantitative analysis of pa-AL numbers in the CA1 region of mouse hippocampus based on staining data in (**c**) (*n* = 30 fields from three mice; ***P = 0.0001, ****P < 0.0001). **e–g** Immunoblot analysis of P62 and LC3B-II/I in the hippocampi of NonTg, 3 × Tg, and 3 × Tg-*Ndst3*^+/−^ mice (*n* = 3 independent experiments; *P = 0.0269, **P < 0.01, ****P < 0.0001). **h–j** Immunoblotting analysis of APP-CTFβ (6E10 antibody) and MAPT/tau in the hippocampi (*n* = 3 independent experiments; *P < 0.05, **P = 0.0053, ***P = 0.0008). **k**, **l** Immunohistochemical staining and quantification of Aβ plaques (6E10 antibody) in mouse hippocampi (Scale bar, 100 μm;* n* = 3 mice; *P = 0.0144, **P = 0.0020). **m**, **n** Immunohistochemical staining and quantification of p-MAPT/tau (Ser422) in mouse hippocampi (Scale bar, 100 μm; *n* = 3 mice; *P = 0.0118, **P = 0.0021). **o**, **p** Thioflavin-S staining of amyloid plaques in mouse hippocampi (Scale bar, 100 μm; *n* = 3 mice; ***P < 0.001). CA1 and DG subregions were combined for quantification of hippocampal immunohistochemical staining and Thioflavin-S staining analyses. Error bars represent SEMs. One-way ANOVA followed by Dunnett’s post hoc tests

To further explore the role of NDST3 in lysosomes in vivo, we investigated whether the suppression of NDST3 expression enhances lysosomal function and subsequent Aβ and MAPT/tau degradation in 3 × Tg-AD mice. Using 3 × Tg-AD mice, we generated a new mouse line with genetic downregulation of NDST3 expression. First, we generated heterozygous *Ndst3* knockout (*Ndst3*^*+/−*^) mice using CRISPR/Cas9 technology and crossbred them to obtain homozygous *Ndst3* knockout (*Ndst3*^*−/−*^*)* mice (Fig. S6a, b). Consistent with previous literature [44], *Ndst3*^*−/−*^ mice were viable, fertile, and exhibited no overt alterations in blood biochemical analyses for renal function (UREA and CREA), hepatic function (ALT and AST), or metabolic profile (GLU, TG, TC, HDL-C, and LDL-C) (Fig. S6c–k). We then crossbred the *Ndst3*^*−/−*^ mice with 3 × Tg-AD mice for several generations, ultimately obtaining a mouse strain exhibiting both the AD phenotype and downregulation of NDST3 expression (Fig. S6l). Analysis of the genetic background of the mice validated the generation of 3 × Tg-*Ndst3*^*+/−*^ mice (Fig. S6m). The western blot results revealed a significant reduction in NDST3 protein level in the hippocampi of 3 × Tg-*Ndst3*^*+/−*^ mice compared with the control 3 × Tg-AD mice (Fig. 5a, b). The 3 × Tg-*Ndst3*^*+/−*^ mice showed no noticeable differences in general health, feeding behavior, or survival relative to their littermate controls during the study period. Blood biochemical analysis also revealed no signs of major organ toxicity in the 3 × Tg-*Ndst3*^*+/−*^ mice (Fig. S6c–k).

Consistent with the results in HT22 cells, immunofluorescence analysis revealed that perinuclear microtubule acetylation was significantly reduced in the hippocampal CA1 and DG regions of 3 × Tg-AD mice compared to NonTg controls; however, it was restored in the 3 × Tg-*Ndst3*^*+/−*^ mice (Fig. S7a–d).

We next sought to validate the restoration of lysosomal acidification in vivo. We introduced a tandem mCherry-eGFP-MAP1LC3/LC3 construct driven by the neuron-specific promoter hSyn into the brains of 9-month-old 3 × Tg-*Ndst3*^*+/−*^ mice, as well as age-matched NonTg and control 3 × Tg-AD mice, via AAV-mediated delivery into the lateral ventricle. This tandem fluorescence-tagged LC3 (tfLC3) exhibits yellow fluorescence at the neutral pH of autophagosomes. Upon fusion with lysosomes and the formation of autolysosomes (ALs), the color of the tfLC3 fluorescence signal shifts from yellow to orange and finally to red as eGFP fluorescence is gradually quenched at pH values below 6.0. Combined with fluorescence staining for a lysosomal marker such as CTSB tagged with a third fluorophore (in this study, Alexa Fluor 647, pseudocolored blue), we could differentiate fully acidified ALs (f-ALs) from poorly acidified ALs (pa-ALs) [21]. As shown in Fig. 5c, d, f-ALs appeared purple, whereas pa-ALs appeared white. tfLC3 was fully expressed in the hippocampus one month after injection into the brain. Compared with age-matched NonTg controls, the 10-month-old 3 × Tg-AD mice presented a significant increase in the number of pa-ALs. Notably, this increase was substantially reversed in age-matched 3 × Tg-*Ndst3*^*+/−*^ mice (Fig. 5c, d), indicating that reduced NDST3 expression promotes lysosomal acidification. In addition, compared to NonTg mice, 3 × Tg-AD mice displayed markedly increased conversion of LC3B-I to LC3B-II (a hallmark of autophagic induction or suppression at the late stage) and accumulation of P62 (indicating autophagic suppression). In contrast, the 3 × Tg-*Ndst3*^*+/−*^ mice exhibited significant reductions of both the LC3B-II/LC3B-I ratio and the P62 level compared to age-matched 3 × Tg-AD mice (Fig. 5e–g).

We further explored whether the restoration of lysosomal acidification upon decreased NDST3 expression leads to reduced Aβ and MAPT/tau burden in the hippocampus of 3 × Tg-AD mice. Consistent with previous research [45–47], western blot analysis showed elevated hippocampal levels of APP-CTFβ (the direct precursor of Aβ) and MAPT/tau in the 10-month-old 3 × Tg-AD mice, which were, however, significantly reduced in the age-matched 3 × Tg-*Ndst3*^*+/−*^ mice (Fig. 5h–j). To directly visualize the downstream pathological consequences of this reduced precursor load, we performed immunostaining for Aβ plaques and phosphorylated MAPT/tau. Given that these pathologies initially develop in the caudal hippocampus of 3 × Tg-AD mice [46], we analyzed this specific region using antibodies against Aβ and p-MAPT/tau (Ser422), an early marker for tangle formation. As expected, the NonTg mice showed almost no immunoreactivity for these markers. In contrast, 3 × Tg-AD mice displayed abundant Aβ plaques and p-MAPT/tau (Ser422) aggregates. Importantly, 3 × Tg-*Ndst3*^*+/−*^ mice exhibited a significant reduction in both Aβ plaque number and p-MAPT/tau (Ser422) immunoreactivity compared to 3 × Tg-AD mice (Fig. 5k–n). The reduced amyloid pathology in 3 × Tg-*Ndst3*^+/-^ mice was further validated by thioflavin-S staining (Fig. 5o, p).

### Decreased NDST3 expression ameliorates neuronal impairment in 3 × Tg-AD mice

**Fig. 6:**
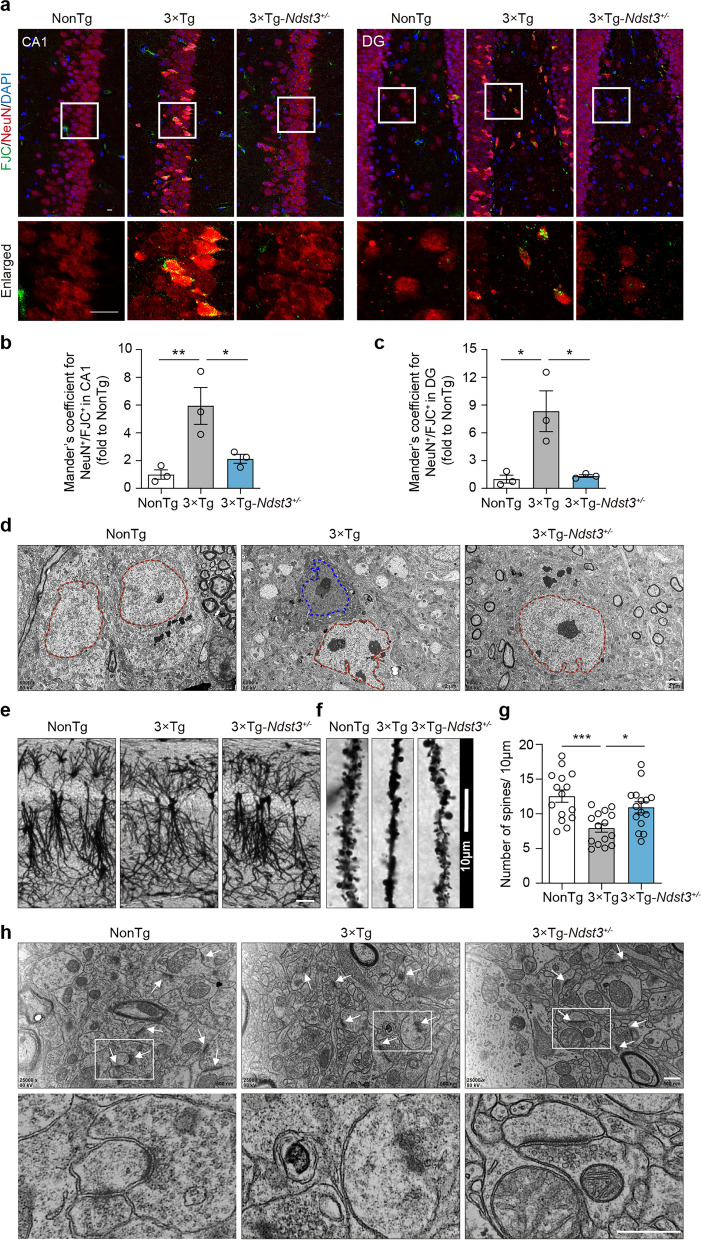
Decreased NDST3 expression ameliorates neuronal impairment in 10-month-old 3 × Tg-AD mice. **a** Representative images of Fluoro-Jade C (FJC) and NeuN double labeling in the mouse hippocampus. Scale bars, 10 μm. **b**,** c** Quantification of FJC signals in NeuN-positive cells using Mander’s coefficients in hippocampal CA1 (**b**) and DG (**c**) (*n* = 3 mice per group; *P < 0.05, **P = 0.0090). **d** Transmission electron microscopy (TEM) images depicting neuronal morphology. The dashed lines outline the nucleus, and the blue dashed lines indicate dark neurons. Scale bar, 2 μm. **e**,** f** Representative images of Golgi staining of dendrites (**e**) and dendritic spines (**f**) in mouse hippocampal CA1 region. Scale bars, 50 μm (**e**), 10 μm (**f**). **g** Quantitative analysis of the number of dendritic spines based on the Golgi staining data in (**f**) (*n* = 15 neurons from three mice; *P = 0.0170, ***P = 0.0003). **h** High-resolution TEM images depicting the fine structures of neurons in the mouse hippocampus. The white arrows indicate synapses. Scale bars, 500 nm. Error bars represent SEMs. One-way ANOVA followed by Dunnett’s post hoc tests

Reductions of the Aβ and MAPT/tau burdens in neurons may contribute to improved neuronal function and survival [48]. The increased cell survival of APP695^Swe^- and MAPT/tau^P301L^-overexpressing HT22 cells following sh*Ndst3* treatment provided preliminary in vitro evidence for the beneficial effects of reducing the Aβ and MAPT/tau burden (Fig. S8a, b). Next, we evaluated whether decreased NDST3 expression can ameliorate neuronal impairment in vivo. Nissl staining revealed impaired neuronal morphology and reduced neuronal density in the hippocampi of 3 × Tg-AD mice compared with the NonTg mice. However, compared with 3 × Tg-AD mice, the 3 × Tg-*Ndst3*^*+/−*^ mice presented preserved neuronal morphology and increased cell density (Fig. S8c, d). To specifically identify degenerating neurons, we performed co-immunostaining of FJC and the neuronal marker NeuN in the hippocampal sections of NonTg, 3 × Tg-AD, and 3 × Tg-*Ndst3*^*+/−*^ mice. Quantification of colocalization using Mander’s coefficients revealed a significantly greater overlap between FJC^+^ and NeuN^+^ signals in both the hippocampal CA1 and DG regions of 3 × Tg-AD mice compared to the NonTg controls. However, decreased NDST3 expression in 3 × Tg-*Ndst3*^*+/−*^ mice significantly attenuated these colocalizations compared to 3 × Tg-AD mice (Fig. 6a–c). Similarly, TEM revealed abnormalities in the neuronal nuclear volume, nuclear membrane folding, and chromatin marginalization, as well as the presence of dark neurons, in 3 × Tg-AD mice. In contrast, after NDST3 expression was downregulated in 3 × Tg-AD mice, these changes were notably reversed (Fig. 6d).

To elucidate the intricate details of the neuronal architecture, Golgi staining and high-resolution TEM were utilized to examine the fine structure of neurons, including the soma, dendrites, dendritic spines, and synapses. The Golgi staining results revealed that dendrites were sparser and that the density of mature spines was lower in the 3 × Tg-AD mice than in the NonTg mice. Reduction of NDST3 expression rescued these dendritic and dendritic spine abnormalities (Fig. 6e–g). Furthermore, TEM revealed that in the 3 × Tg-AD mice, the presynaptic and postsynaptic membranes were fused, the synaptic cleft was blurred, and synaptic vesicles were sparse and disordered. Reduction of NDST3 expression restored the structure of synapses and the number of synaptic vesicles to be similar to those in NonTg mice (Fig. 6h).

Interestingly, in addition to improving neuronal health, we observed that NDST3 knockdown also significantly modulated the innate immune response. Immunostaining for Iba1 revealed a significant increase in total microglial load in the hippocampus of 3 × Tg-AD mice relative to the NonTg controls, whereas this increase was reversed in 3 × Tg-*Ndst3*^*+/−*^ mice (Fig. S9a, b). Activated microglia, indicated by colocalization of CD68 and Iba1 signals, were also markedly increased in 3 × Tg-AD mice compared to NonTg controls; however, this pathological activation was attenuated in 3 × Tg-*Ndst3*^*+/−*^ mice (Fig. S9c–e). In contrast to the microglial response, the hippocampal GFAP^+^ area was significantly decreased in 3 × Tg-AD mice compared to the NonTg controls, indicative of astrocytic atrophy or functional exhaustion in the AD model. Notably, downregulation of NDST3 significantly restored GFAP expression in 3 × Tg-*Ndst3*^*+/−*^ mice compared to the 3 × Tg-AD littermates (Fig. S9f, g).

### Decreased NDST3 expression ameliorates cognitive deficits in 3 × Tg-AD mice

**Fig. 7:**
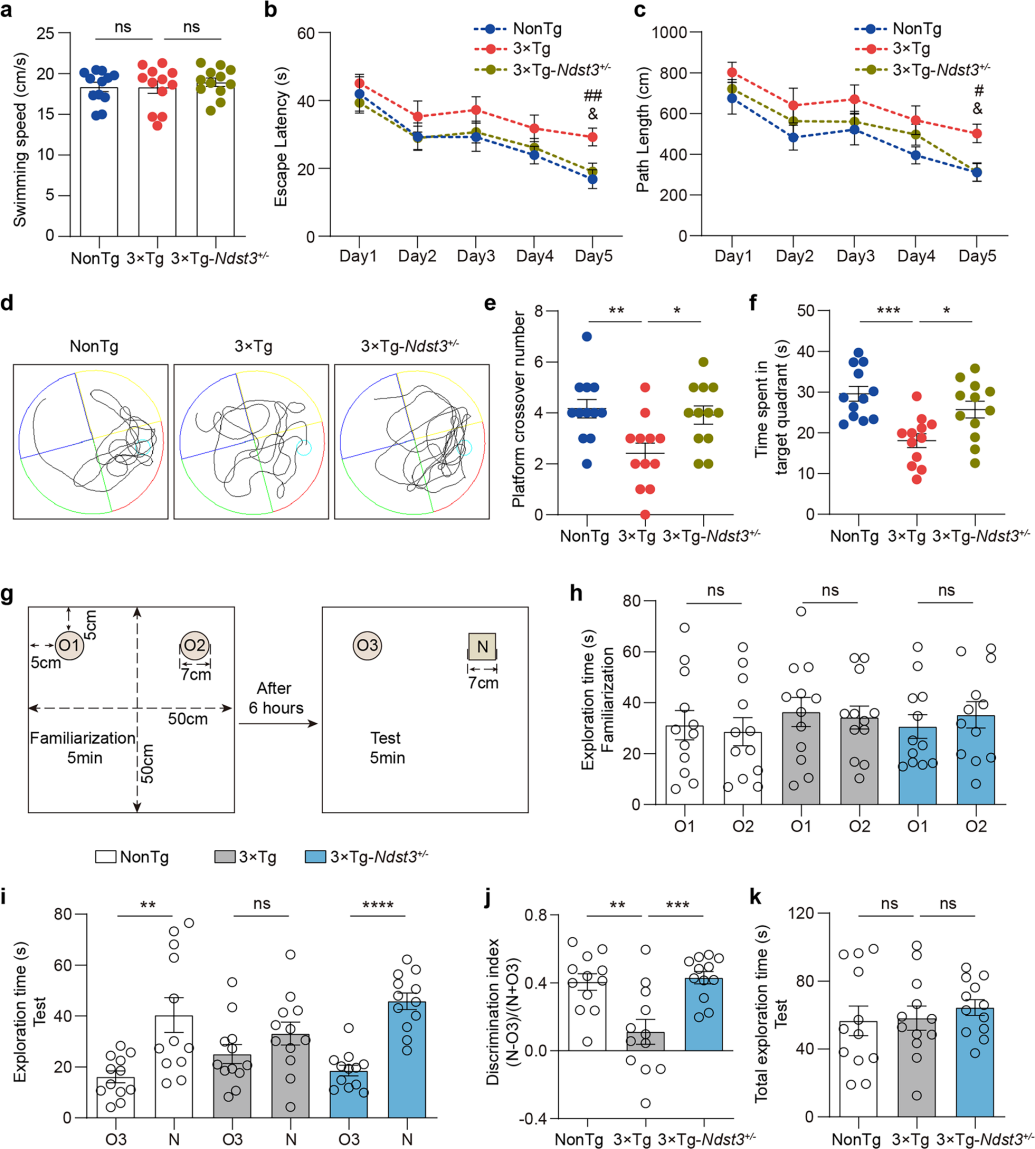
Decreased NDST3 expression ameliorates cognitive deficits in 10-month-old 3 × Tg-AD mice. **a** Average swimming speed of mice during the adaptation period prior to training in the Morris water maze test (*n* = 12 mice per group; ns represents nonsignificant). **b**,** c** The average latency (**b**) and swimming distance (**c**) to locate the hidden platform during the spatial learning phase (days 1–5) (*n* = 12 mice per group; ^*#*^*P* = 0.0156 and ^*##*^*P* = 0.0099 for 3 × Tg vs. NonTg, ^*&*^*P* < 0.05 for 3 × Tg-*Ndst3*^+/−^ mice vs. 3 × Tg mice). **d** Representative swimming traces of mice during the probe phase. **e**,** f** Numbers of platform location crossings (**e**) and time spent in the target quadrant (**f**) during the probe phase (*n* = 12 mice per group; *P < 0.05, **P = 0.0044, ***P = 0.0002). **g** Schematic diagram of the novel object recognition test (NOR) procedure, consisting of familiarization and test phases. O1 and O2 denote identical objects; O3 is an object identical to O1, and N is a novel object with a similar size as O1 but distinct in shape and color. **h** Time spent exploring O1 and O2 during the familiarization phase of the NOR (*n* = 12 mice per group; ns represents nonsignificant). **i** Time spent exploring novel (N) and familiar (O3) objects during the testing phase of NOR (*n* = 12 mice per group; **P = 0.0030, ****P < 0.0001, ns represents nonsignificant). **j** Discrimination index during the NOR test phase was calculated from data in (**h**) and (**i**) using the equation described in *Materials and Methods* (*n* = 12 mice per group; **P = 0.0012, ***P = 0.0005). **k** Total exploration time of O3 and N in the NOR test phase (*n* = 12 mice per group; ns represents nonsignificant). Error bars represent SEMs. One-way ANOVA followed by Dunnett’s post hoc tests was used for statistical analyses in (**a**, **e**, **f**, **j**, and **k**). Two-way repeated-measures ANOVA was used to statistically analyze the data in (**b** and **c**). Two-tailed Student’s t tests (**h** and **i)**

Cognitive impairment, especially learning and memory deficits, is a prominent manifestation of neuronal impairment in 3 × Tg-AD mice aged 6.5 months or older [49]. Considering the beneficial effect of decreased NDST3 expression on neurons, we investigated the ability of NDST3 downregulation to ameliorate cognitive deficits in 3 × Tg-AD mice. Ten-month-old 3 × Tg-*Ndst3*^*+/−*^ mice, NonTg and control 3 × Tg-AD mice were subjected to the Morris water maze and novel object recognition tests to assess spatial memory and recognition memory, respectively. Before the Morris water maze test, swimming speed was compared among the groups of mice to exclude the possibility that any differences were due to variations in swimming speed (Fig. 7a). On the final day of the training phase, the 3 × Tg-AD mice exhibited longer escape latencies and swimming distances than NonTg mice, whereas the 3 × Tg-*Ndst3*^*+/−*^ mice showed significantly shorter latencies and swimming distances than 3 × Tg-AD mice (Fig. 7b, c). During the probe test following the training phase, the 3× Tg-AD mice spent less time swimming in the target quadrant and crossed the platform location fewer times than NonTg mice did. In contrast, compared with 3 × Tg-AD mice, 3 × Tg-*Ndst3*^*+/−*^ mice spent more time in the target quadrant and had more crossings at the platform location (Fig. 7d–f). The observed differences were not attributable to variations in swimming ability, as the swimming speed was similar across all groups (Fig. 7a).

Similar improvements were found in the novel object recognition test. During the familiarization phase, the time spent exploring either of the two identical objects did not differ among the NonTg, 3× Tg-AD, and 3 × Tg-*Ndst3*^*+/−*^ mice (Fig. 7g, h). However, in the testing phase, the NonTg and 3 × Tg-*Ndst3*^*+/−*^ mice, but not the 3 × Tg-AD mice, spent significantly more time exploring the novel object than the familiar object (Fig. 7i), with discrimination indexes reaching  0.4, significantly higher than that of the 3 × Tg-AD mice (Fig. 7j). The total time spent exploring the two objects in the testing phase was not different among NonTg, 3 × Tg-AD, and 3 × Tg-*Ndst3*^*+/−*^ mice, ruling out the interference of overall activity levels (Fig. 7k). These behavioral test results demonstrated that the 3 × Tg-AD mice present significant cognitive deficits, which are notably ameliorated by downregulation of NDST3.

## Discussion

NDST3 is primarily recognized as a bifunctional enzyme catalyzing the N-deacetylation and N-sulfation of N-acetylglucosamine residues in heparan sulfates [44, 50]. Beyond this classical role, our previous investigation [27] found a novel physiological function of NDST3 in lysosomal regulation. We demonstrated that *NDST3* knockout in normal RPE1 cells causes excessive lysosomal acidification. The lysosomal over-acidification is detrimental to lysosomal hydrolase and proteolytic activities, which was proven by our subsequent analysis in *C9orf72*-linked amyotrophic lateral sclerosis (ALS) and other recent works [51–54]. Specifically, in *C9orf72*-linked ALS, C9orf72 haploinsufficiency reduces NDST3 expression (since NDST3 is positively regulated by C9orf72), resulting in lysosomal over-acidification and compromised lysosomal function. In contrast, the present study focuses on AD models, in which insufficient lysosomal acidification is a well-recognized pathological feature [19]. This impaired acidification contributes to key AD pathologies, including Aβ accumulation and tau hyperphosphorylation [21, 55, 56]. NDST3 was upregulated in AD neuronal cells, mouse models, and human patients compared with healthy controls. Although we did not determine whether lysosomal alkalinization and dysfunction in AD are attributed to the upregulation of NDST3, we found that NDST3 knockdown in the AD context restores lysosomal pH toward the physiological range, thereby improving lysosomal function and facilitating the autophagic degradation of aberrant Aβ and MAPT/tau, finally exerting neuroprotective effects. Therefore, our current results in AD are not contradictory to our previous findings in ALS; instead, they reveal a biphasic and context-dependent role for NDST3 in lysosomal pH regulation: in normal cells, NDST3 deficiency induces over-acidification and cellular stress (detrimental effect), whereas in the AD pathology setting, NDST3 downregulation counteracts under-acidification and restores lysosomal homeostasis (protective effect). These disease-specific pathological and molecular features highlight the complexity of neurodegenerative disorders and emphasize the need for tailored research approaches to dissect disease-specific mechanisms and identify novel therapeutic targets.

Previous reports have established that trichostatin A (TSA), a histone deacetylase inhibitor that suppresses HDAC6-mediated microtubule deacetylation, reduces lysosomal pH [29]. Building on this, our recent publications systematically clarified the mechanistic link between microtubule acetylation status and lysosomal acidification, demonstrated via genetic or pharmacological interventions including HDAC6 inhibition, NDST3 ablation, and treatments with tubacin, nocodazole, or paclitaxel [27, 30]. Specifically, we showed that microtubule acetylation retains the V-ATPase V1 subunits and lysosomes in the perinuclear region, thereby promoting the V-ATPase V1-V0 assembly and enhancing proton translocation into the lysosomal lumen [27]. Given that restoring lysosomal acidification is a major therapeutic goal for AD [19, 23, 57], these findings highlight microtubule-targeting strategies as promising avenues. Indeed, HDAC6 is a widely studied therapeutic target for AD [40]. Over the past years, researchers have developed dozens of selective HDAC6 inhibitors for AD treatment [58]. In this study, we report that NDST3 exhibits a comparable capacity to HDAC6 in modulating lysosomal acidification. However, unlike HDAC6, which broadly regulates microtubule deacetylation throughout the cytoplasm, NDST3 primarily exerts its deacetylase activity on perinuclear microtubules. This spatial specificity suggests that NDST3 may represent a more targeted modulator of lysosomal acidification in AD, potentially minimizing the broader cytoskeletal alterations associated with HDAC6 inhibition.

Uncovered by single-cell sequencing profiles [39] and corroborated by our findings in this study, NDST3 is predominantly expressed in the brain and displays a neuron-specific expression pattern. This characteristic distinguishes it from ubiquitously expressed deacetylases and suggests that targeting NDST3 could offer a higher safety profile for AD therapeutics by minimizing systemic off-target effects. Supporting this notion, a previous systematic study on NDST3 KO mice reported that the *Ndst3*^*−/−*^ mice are born at the expected Mendelian ratio, are viable and fertile, and exhibit an overall normal phenotype with the exception of subtle alterations in some hematological parameters and reduced anxiety-related behavior [44]. Furthermore, our serum biochemical analyses revealed no significant differences in liver function, renal function, blood glucose, or lipid metabolism between *Ndst3*^*−/−*^ mice and NonTg controls. In the AD context, we utilized 3 × Tg-AD mice with partial NDST3 KO (3 × Tg-*Ndst3*^*+/−*^ mice) due to logistical challenges associated with the complex breeding scheme required to generate a sufficiently large cohort of complete KO mice for behavioral testing. This partial knockout achieved significant NDST3 downregulation in the hippocampus, which was sufficient to restore lysosomal acidification. Importantly, this intervention did not compromise animal health. 3 × Tg-*Ndst3*^*+/−*^ mice showed no noticeable differences in general health, feeding behavior, or survival compared to littermate controls during the study period. Admittedly, however, our current safety assessment remains preliminary. Future studies will be required to profoundly evaluate the safety profile of NDST3 deficiency, with a specific focus on neurological impact.

Unlike methods involving direct clearance of Aβ and MAPT/tau, such as antibody-based therapies [59, 60], knocking down NDST3 enhances lysosomal degradation within neurons to reduce aberrant Aβ and MAPT/tau levels, thereby mitigating their extracellular accumulation and deposition. This attenuation of Aβ and MAPT/tau burden not only improves neuronal health but also contributes to the amelioration of secondary neuroinflammation in 3 × Tg-*Ndst3*^*+/−*^ mice. This approach targets the upstream pathological cascade of AD rather than merely addressing downstream end products, which may confer more sustainable therapeutic effects by restoring the intrinsic cellular proteostasis machinery. Specifically, lysosomal dysfunction is an early and pivotal event in AD pathogenesis, occurring prior to the overt accumulation of Aβ plaques or exacerbating MAPT/tau pathology [21, 55, 56, 61–63]. Therefore, reactivating lysosomal function via NDST3 knockdown allows interception of the disease process at its initiation stage. Moreover, since this intervention acts by rectifying endogenously dysregulated pathways, it is likely more compatible with the complex brain microenvironment, minimizing the risk of unintended disruptions to normal neuronal function.

This study has several limitations that point to important future research directions. First, we lack longitudinal data delineating the temporal effects of NDST3 reduction on AD cognitive decline. This limitation stems from the challenges of producing a cohort of sufficient size for statistically robust behavioral analysis due to the complexity and extended timeline of our breeding scheme to generate the 3 × Tg-*Ndst3*^*+/−*^ mice. Second, we have not yet developed specific pharmacological agents targeting NDST3 to restore lysosomal acidification and function, primarily due to the current lack of detailed structural information about this protein. Without a comprehensive understanding of its structure, particularly the active sites involved in the regulation of lysosomal acidification, developing effective pharmacological agents remains a significant challenge. Additionally, two potential caveats associated with the experimental systems employed in this study should be acknowledged. For the in vivo lysosomal pH determination, AAV-mediated delivery of hSyn-mCherry-GFP-LC3B into the brain could result in LC3B overexpression, a factor that may affect the interpretation of experimental results. However, we have minimized such effects by standardizing injection parameters across all groups and conducting intergroup comparisons of the results. Furthermore, in our in vitro HT22 cell experiments, we used an APP695^Swe^ overexpression model where endogenous APP remains intact (without knockout of the endogenous APP gene); thus, this system is accurately characterized as “HT22 cells with APP695^Swe^ overexpression” rather than an “HT22 cell model of AD". Therefore, establishing longitudinal behavioral cohorts to clarify the temporal effects of NDST3 reduction on AD-related cognitive decline, elucidating the NDST3 structure to identify druggable pockets for developing targeted pharmacological agents, and validating our findings using more models relevant to clinical AD pathogenesis will be the focus of our future work. These efforts are critical for advancing NDST3-targeted therapeutic strategies for AD.

## Conclusions

**Fig. 8:**
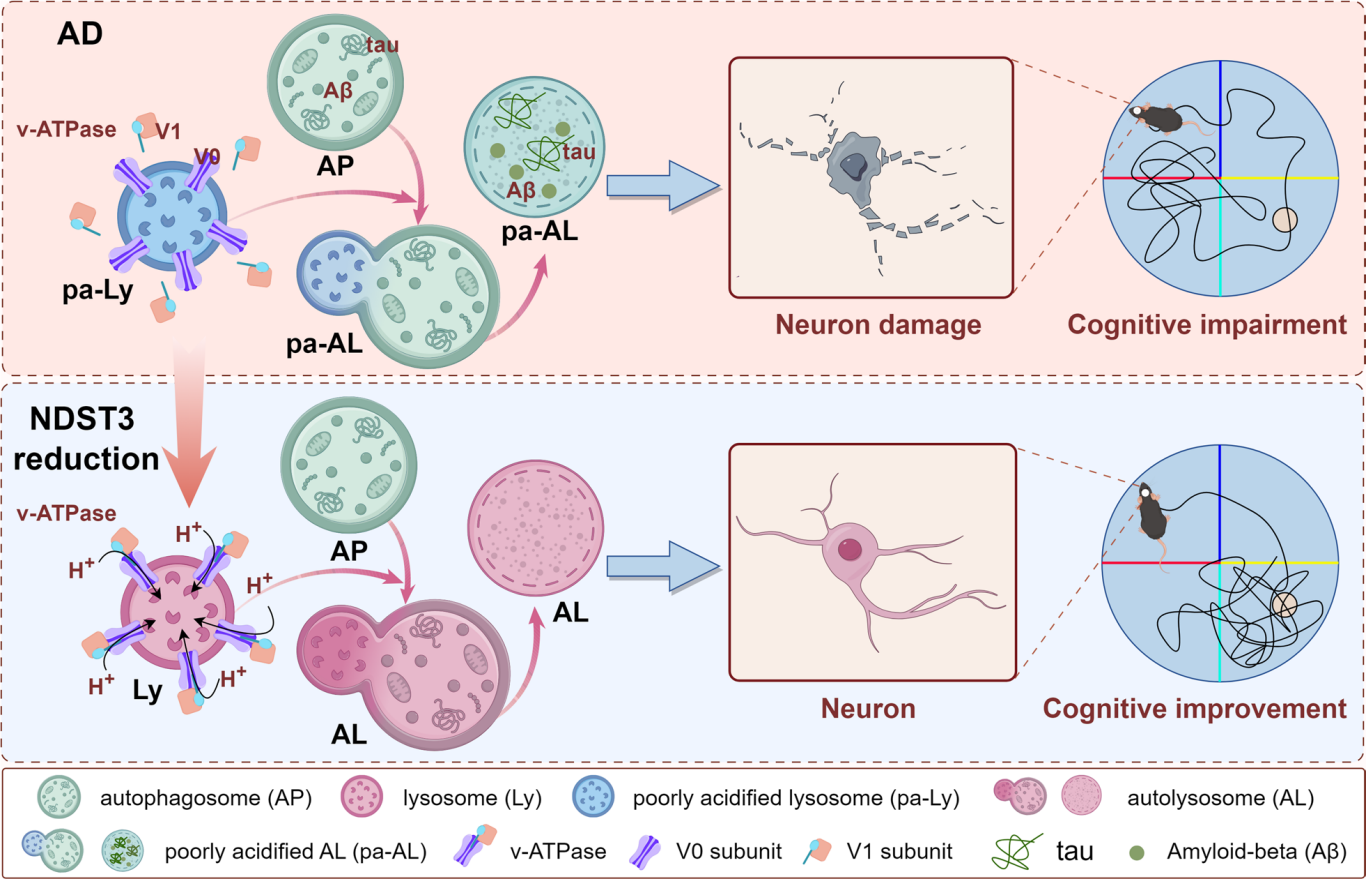
Schematic model of the role of NDST3 suppression in ameliorating AD pathology. NDST3 suppression enhances the assembly of the V-ATPase V1 and V0 domains on the lysosomal membrane, restoring lysosomal acidification in neurons and increasing the lysosomal degradation of autophagic substrates such as aberrant Aβ and MAPT/tau in AD models. Timely degradation of these toxic proteins reduces neuronal damage in the brains of AD model mice, facilitating the recovery of cognitive function. Figure was created with Figdraw

In this study, we report a therapeutic strategy to mitigate the Aβ and MAPT/tau burden in AD by restoring lysosomal acidification and function via the targeting of NDST3, a previously unknown regulator of lysosomal acidification in AD. Suppression of NDST3 restores lysosomal degradation of aberrant Aβ and MAPT/tau, thereby reducing their accumulation and deposition, preventing neuronal damage, and ameliorating cognitive deficits in the 3 × Tg-AD mouse model (Fig. 8). The development of NDST3-targeted therapies represents a promising direction for future research on AD and possibly other neurodegenerative diseases.

## Supplementary Information

Additional file 1. **Figure S1.** NDST3 and HDAC6 functional comparison, knockout validation, and NDST3 localization. **Figure S2.** Validation of HT22-APP695^Swe^ model, lysosomal isolation and V-ATPase subunit expression in NDST3-knockdown whole-cell lysates. **Figure S3.** Nucleus-associated LAMP2, total LAMP2, and LAMP2-CTSB colocalization in NDST3-knockdown APP695^Swe^-overexpressing cells. **Figure S4.** Autophagic degradation of p-MAPT/tau (Ser262) and p-MAPT/tau (Thr212) in HT22 cells with NDST3 knockdown. **Figure S5.** NDST3 expression in 3- and 12-month-old 3xTg-AD mouse brain, AD patient characteristics, and lysosomal pH in NDST3-overexpressing models. **Figure S6.** *Ndst3* KO and 3xTg-*Ndst*^3+/-^ mouse generation, genotyping and blood biochemical analyses. **Figure S7.** Perinuclear microtubule acetylation in the hippocampi of 10-month-old 3xTg-*Ndst*^3+/-^ mice and age-matched NonTg and 3xTg-AD controls. **Figure S8.** Cell survival in APP695^Swe^- or MAPT/tau^P301L^-overexpressing HT22 cells with NDST3 knockdown, and Nissl staining in the hippocampus of NonTg, 3xTg, and 3xTg-*Ndst*^3+/-^ mice. **Figure S9.** Microglia and astrocyte activation in the hippocampi of 10-month-old NonTg, 3xTg-AD, and 3xTg-*Ndst*^3+/-^ mice.

Additional file 2. Uncropped gels and blots.

## Data Availability

The data supporting the findings of this study are available within the article and/or its supplementary materials.

## Abbreviations

Aβ: Amyloid-β
AD: Alzheimer's disease
AIF1/Iba1: Allograft inflammatory factor 1
ALs: Autolysosomes
ALT: Alanine aminotransferase
APP: Amyloid beta precursor protein
AST: Aspartate aminotransferase
Baf A1: Bafilomycin A1
CQ: Chloroquine
CREA: Creatinine
CTSB: Cathepsin B
ELISA: Enzyme-linked immunosorbent assay
f-ALs: Fully acidified ALs
FJC: Fluoro-Jade C
GFAP: Glial fibrillary acidic protein
GLU: Glucose
GOLGA2/GM130: Golgin A2
HDAC6: Histone deacetylase 6
HDL-C: High-density lipoprotein cholesterol
LAMP2: Lysosomal associated membrane protein 2
LDL-C: Low-density lipoprotein cholesterol
MAP1LC3/LC3: Microtubule associated protein 1 light chain 3
MAPT/tau: Microtubule associated protein tau
MBP: Myelin basic protein
NDST3: N-deacetylase and N-sulfotransferase 3
NFTs: Neurofibrillary tangles
NonTg: Nontransgenic
pa-ALs: Poorly acidified ALs
p-MAPT/tau: Phosphorylated microtubule associated protein tau
RBFOX3/NeuN: RNA binding fox-1 homolog 3
TC: Total cholesterol
TEM: Transmission electron microscopy
tfLC3: Tandem fluorescence-tagged microtubule associated protein 1 light chain 3
TG: Triglycerides
UREA: Urea
V-ATPase: Vacuolar-type H^+^-ATPase
VDAC1: Voltage dependent anion channel 1
WT: Wild-type
