# Supplementary material for: NDST3 suppression restores lysosomal acidification and ameliorates amyloid-β and MAPT/tau pathology in Alzheimer’s disease
Source: Transl Neurodegener. 2026 Apr 21;15:16. doi: 10.1186/s40035-026-00549-1 (PMC13097816; doi:10.1186/s40035-026-00549-1)
Supplement: Supplementary file 1 — Additional file 1. Figure S1. NDST3 and HDAC6 functional comparison, knockout validation, and NDST3 localization. Figure S2. Validation of HT22-APP695Swe model, lysosomal isolation and V-ATPase subunit expression in NDST3-knockdown whole-cell lysates. Figure S3. Nucleus-associated LAMP2, total LAMP2, and LAMP2-CTSB colocalization in NDST3-knockdown APP695Swe-overexpressing cells. Figure S4. Autophagic degradation of p-MAPT/tau (Ser262) and p-MAPT/tau (Thr212) in HT22 cells with NDST3 knockdown. Figure S5. NDST3 expression in 3- and 12-month-old 3xTg-AD mouse brain, AD patient characteristics, and lysosomal pH in NDST3-overexpressing models. Figure S6. Ndst3 KO and 3xTg-Ndst3+/- mouse generation, genotyping and blood biochemical analyses. Figure S7. Perinuclear microtubule acetylation in the hippocampi of 10-month-old 3xTg-Ndst3+/- mice and age-matched NonTg and 3xTg-AD controls. Figure S8. Cell survival in APP695Swe- or MAPT/tauP301L-overexpressing HT22 cells with NDST3 knockdown, and Nissl staining in the hippocampus of NonTg, 3xTg, and 3xTg-Ndst3+/- mice. Figure S9. Microglia and astrocyte activation in the hippocampi of 10-month-old NonTg, 3xTg-AD, and 3xTg-Ndst3+/- mice. [file 40035_2026_549_MOESM1_ESM.pdf]

Supplementary Information for

NDST3 suppression restores lysosomal acidification and ameliorates amyloid-β and MAPT/tau pathology in Alzheimer's disease

Inventory of Supplementary Information:

Figure S1-S9 (Page 2-16)

Table S1-S2 (Page 17)

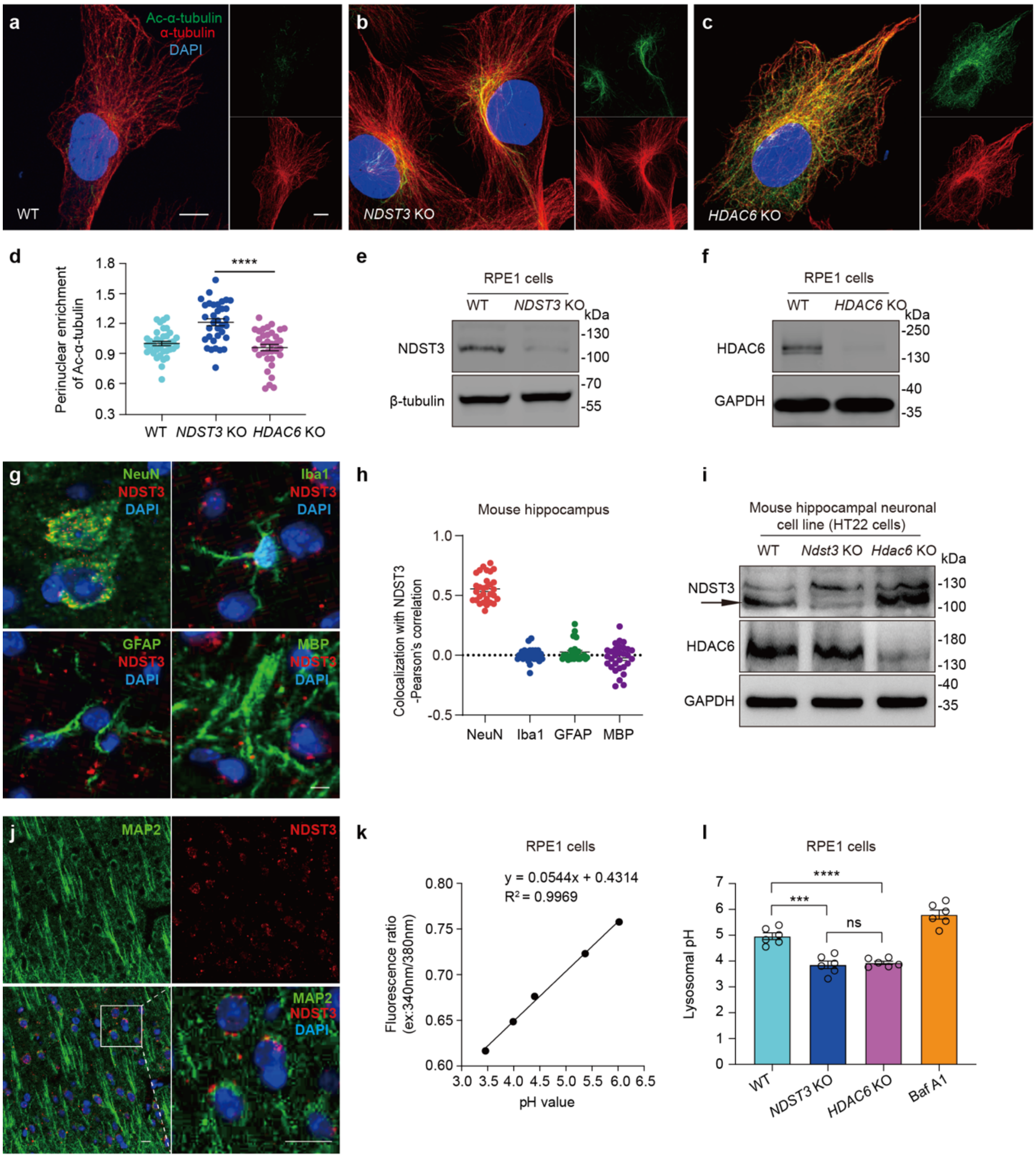

Figure S1. NDST3 and HDAC6 functional comparison, knockout validation, and NDST3 localization. **a**-**c** Representative immunofluorescence staining of acetylated-α-tubulin (Ac-α-tubulin) and total α-tubulin in wild-type (WT) (**a**), *NDST3* KO (**b**) and *HDAC6* KO (**c**) RPE1 cells. Scale bar, 10 μm (main panels and insets). **d** Perinuclear enrichment of Ac-α-tubulin (5-μm perinuclear region, intensity ratio) calculated from (**a**-**c**) (n = 37 cells, WT; n = 34 cells, *NDST3* KO & *HDAC6* KO; ****$P < 0.001$). **e**, **f** Immunoblot analysis of NDST3 and HDAC6 in *NDST3* KO and *HDAC6* KO RPE1 cells, respectively. β-tubulin and GAPDH served as loading controls. **g**, **h** Co-immunofluorescence staining of NDST3 with the

neuronal marker NeuN, microglial marker Iba1, astrocyte marker GFAP, and oligodendrocyte marker MBP in mouse cortex. Pearson's correlation coefficient between NDST3 and each marker was quantified via Fiji (n = 30 cells per group). Scale bar, 5 μm. **i** Immunoblot analysis of NDST3 and HDAC6 in *Ndst3* KO and *Hdac6* KO HT22 cells. GAPDH served as a loading control. **j** Co-immunofluorescence staining of NDST3 with neuronal marker MAP2 in mouse cortex. Scale bar, 10 μm. **k** Calibration curve for lysosomal pH measurement in RPE1 cells (n = 6 independent cultures). **l** Lysosomal pH value calculated from the fluorescence ratio measured by LysoSensor™ yellow/blue DND-160 staining on the basis of the pH calibration curve in (**k**) (n = 6 independent cultures; ***$P = 0.0002$, ****$P < 0.0001$, ns represents nonsignificant). Bafilomycin A1 (Baf A1) served as a positive control. The error bars represent the SEMs. Two-tailed Student's t tests were used for statistical analyses.

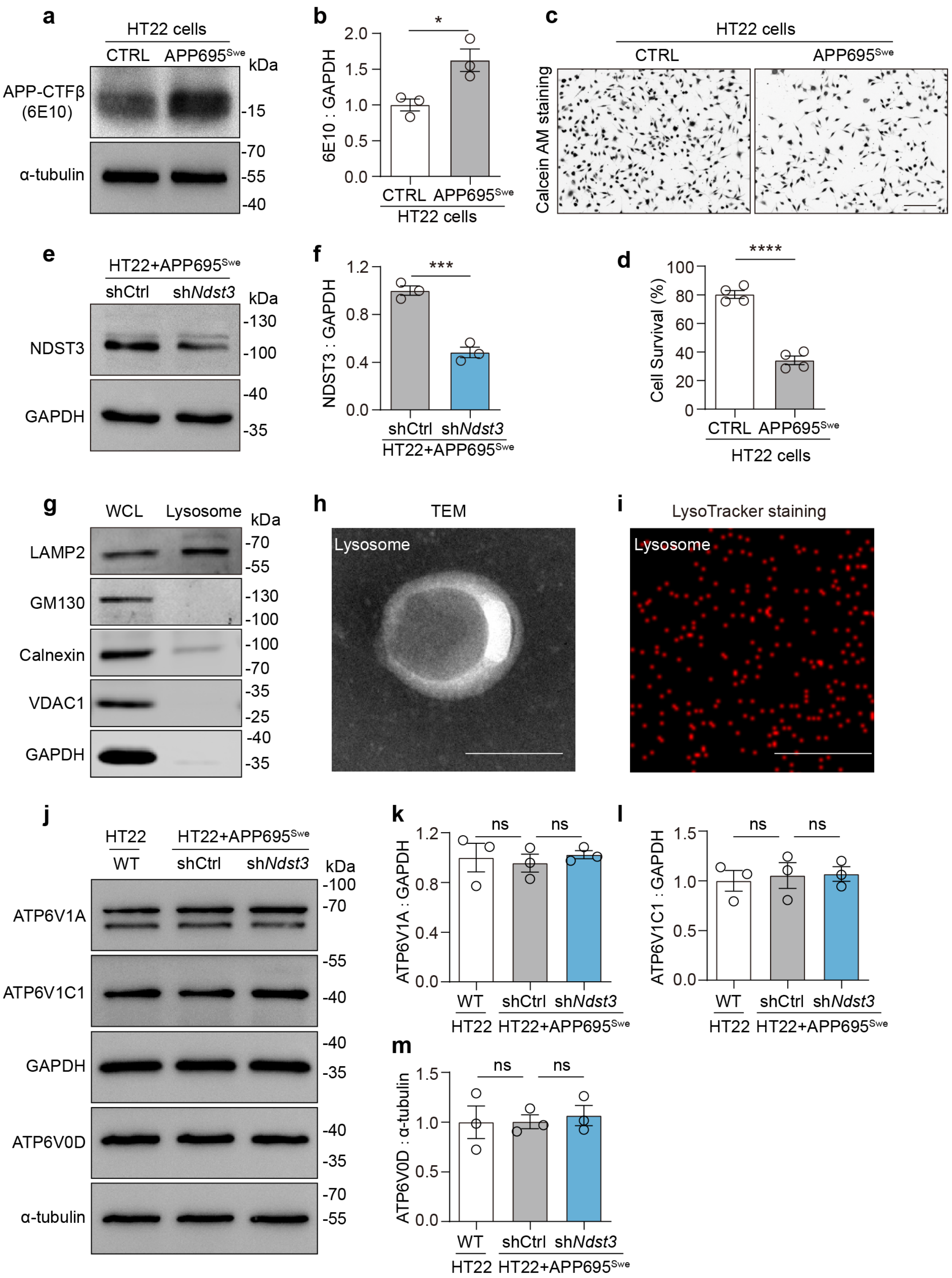

Figure S2. Validation of HT22-APP695^{Swe} model, lysosomal isolation and V-ATPase subunit expression in NDST3-knockdown whole-cell lysates. **a**, **b** Immunoblot analysis of APP-CTFβ (6E10 antibody) in HT22 cells transduced with APP695^{Swe} or control plasmid (CTRL) (*n* = 3 independent experiments; **P* = 0.0249). **c**, **d** Comparative analysis of cell survival in APP695^{Swe}-overexpressing or CTRL HT22 cells, measured by calcein AM

staining at 48 h post-transfection and quantified as a percentage of calcein AM fluorescence intensity relative to untreated positive control cells (n = 4 independent cultures; ****P < 0.0001). Scale bar, 100 μm. **e**, **f** Validation of NDST3 knockdown in HT22 APP695^{Swe} cells with sh*Ndst3* treatment (n = 3 independent experiments; ***P = 0.0009). Error bars represent ± SEMs. Two-tailed Student's t-tests are used for the statistical analyses. **g** Validation of lysosomal isolation by immunoblot analysis for lysosomal marker LAMP2, Golgi marker GM130, endoplasmic reticulum marker Calnexin, mitochondria marker VDAC1, and cytoplasmic marker GAPDH. **h** Transmission electron microscopy (TEM) observation of isolated lysosomes. Scale bar, 100 nm. **i** LysoTracker staining for isolated lysosomes. Scale bar, 5 μm. **j**-**m** Immunoblot analysis (**g**) and quantification of ATP6V1A (**k**), ATP6V1C1 (**l**), and ATP6V0D (**m**) levels in whole-cell lysates of control and APP695^{Swe}-overexpressing cells transfected with shCtrl and sh*Ndst3* (n = 3 independent experiments; ns represents nonsignificant). GAPDH and α-tubulin were used as loading controls.

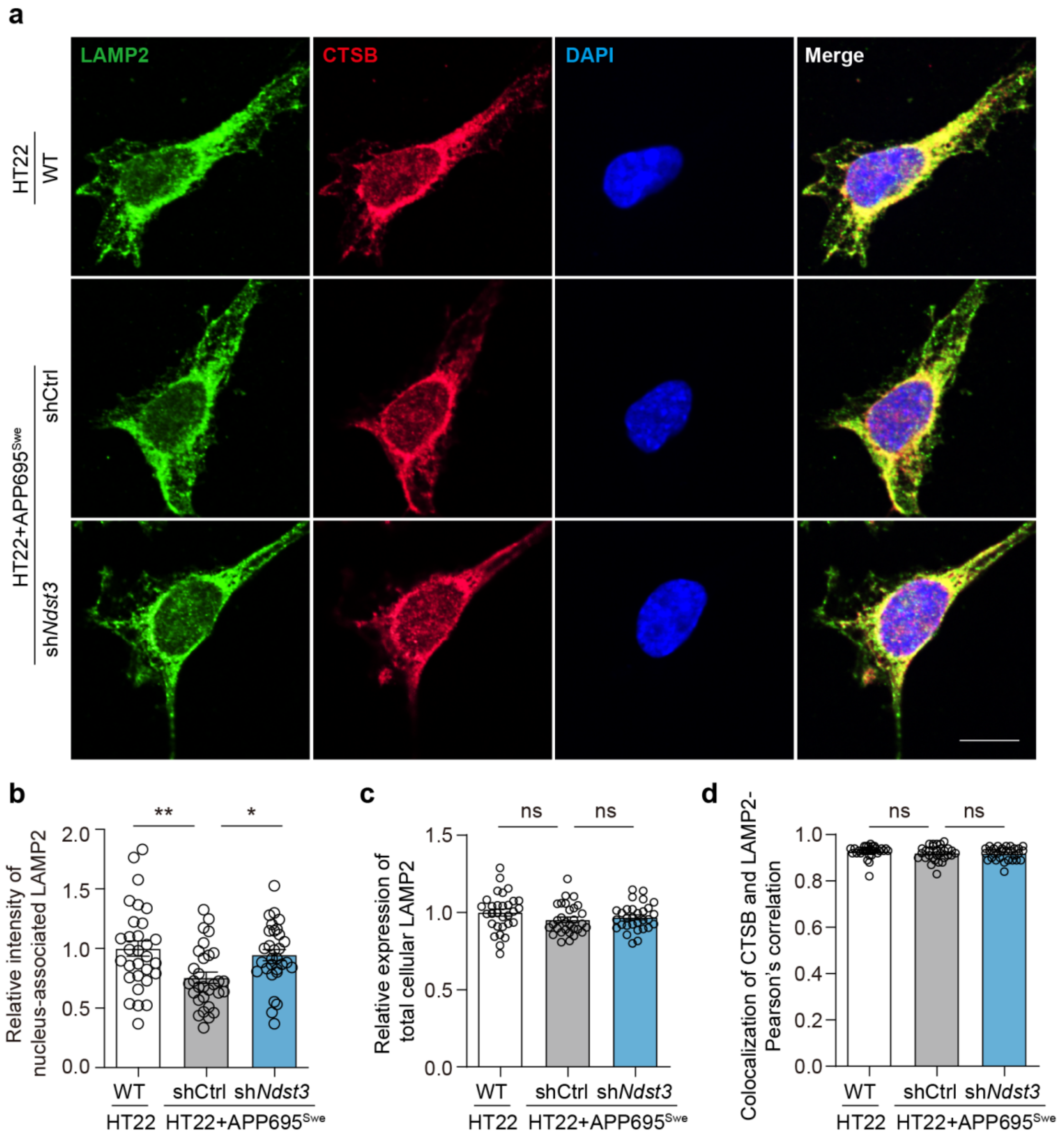

Figure S3. Nucleus-associated LAMP2, total LAMP2, and LAMP2-CTSB colocalization in NDST3-knockdown APP695Swe-overexpressing cells. **a** Co-immunofluorescence staining of lysosomal marker LAMP2 and CTSB. Scale bar, 10 μm. **b** Quantification of LAMP2 signal intensity in the nucleus-associated region (within 5 μm of the DAPI-stained nucleus; *n* = 30 cells per group; **P* = 0.0264, ***P* = 0.0033). **c** Quantification of total cellular LAMP2 intensity relative to WT HT22 cells (*n* = 30 cells per group; ns represents non-significant). (**d**) Colocalization of CTSB with LAMP2 quantified by Pearson's correlation coefficient (*n* = 30 cells per group; ns represents non-significant). **d** Error bars represent ± SEMs. One-way ANOVA followed by Dunnett's post hoc tests are used for the statistical analyses.

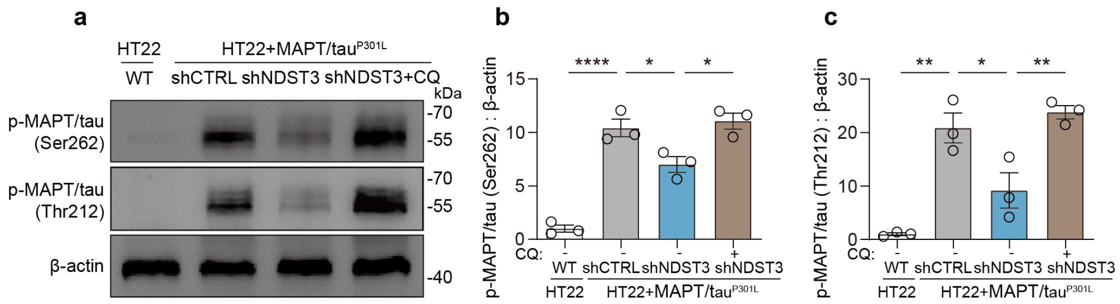

Figure S4. Autophagic degradation of p-MAPT/tau (Ser262) and p-MAPT/tau (Thr212) in HT22 cells with NDST3 knockdown. **a**-**c** Immunoblot analysis of p-MAPT/tau (Ser262) and p-MAPT/tau (Thr212) (n = 3 independent experiments; $*P < 0.05$, $**P < 0.01$, $****P < 0.0001$). β-Actin was used as a loading control. The error bars represent the SEMs. Statistical analyses were performed via one-way ANOVA with Tukey's post hoc tests.

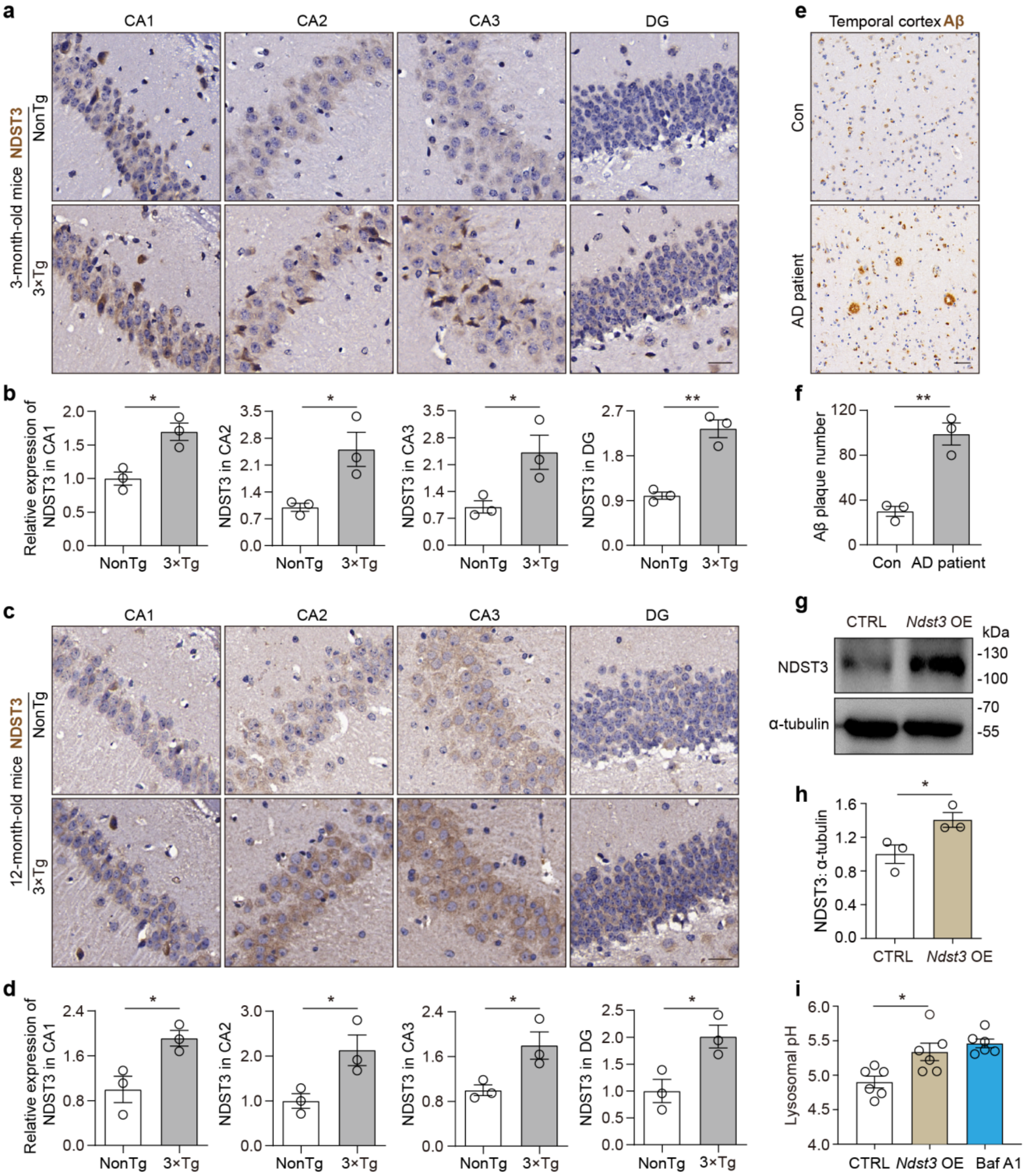

Figure S5. NDST3 expression in 3- and 12-month-old 3xTg-AD mouse brain, AD patient characteristics, and lysosomal pH in NDST3-overexpressing models. **a**, **b** Immunohistochemical staining and quantification of NDST3 in hippocampal CA1, CA2, CA3 and DG regions of 3-month-old 3xTg-AD mice and non-transgenic (NonTg) mice (Scale bar, 25 μm; *n* = 3 mice per group; $^{*}P < 0.05$, $^{**}P = 0.0023$). **c**, **d** Immunohistochemical staining and quantification of NDST3 in hippocampal CA1, CA2, CA3 and DG regions of 12-month-old 3xTg-AD mice and NonTg mice (Scale bar, 25 μm; *n* = 3 mice per group; $^{*}P < 0.05$). **e**, **f** Immunohistochemical staining and quantification of

Aβ plaques in postmortem human brain samples from AD patients and healthy controls. (Scale bar, 50 μm; *n* = 3 human subjects per group; $^{**}P$ = 0.0031). **g**, **h** Immunoblot analysis of NDST3 in HT22 cells transfected with *Ndst3* ORF- containing plasmids (*Ndst3* OE) or vector-only control plasmids (CTRL) (*n* = 3 independent experiments; $^{*}P$ = 0.0444). **i** Lysosomal pH measurement in CTRL and *Ndst3* OE cells via LysoSensor™ yellow/blue DND-160 staining with a pH calibration curve (*n* = 6 independent cultures; $^{*}P$ = 0.0169). Bafilomycin A1 (Baf A1; 100 nM, 10 min) served as a positive control. Error bars represent ± SEMs. Two-tailed Student's t-tests are used for the statistical analyses.

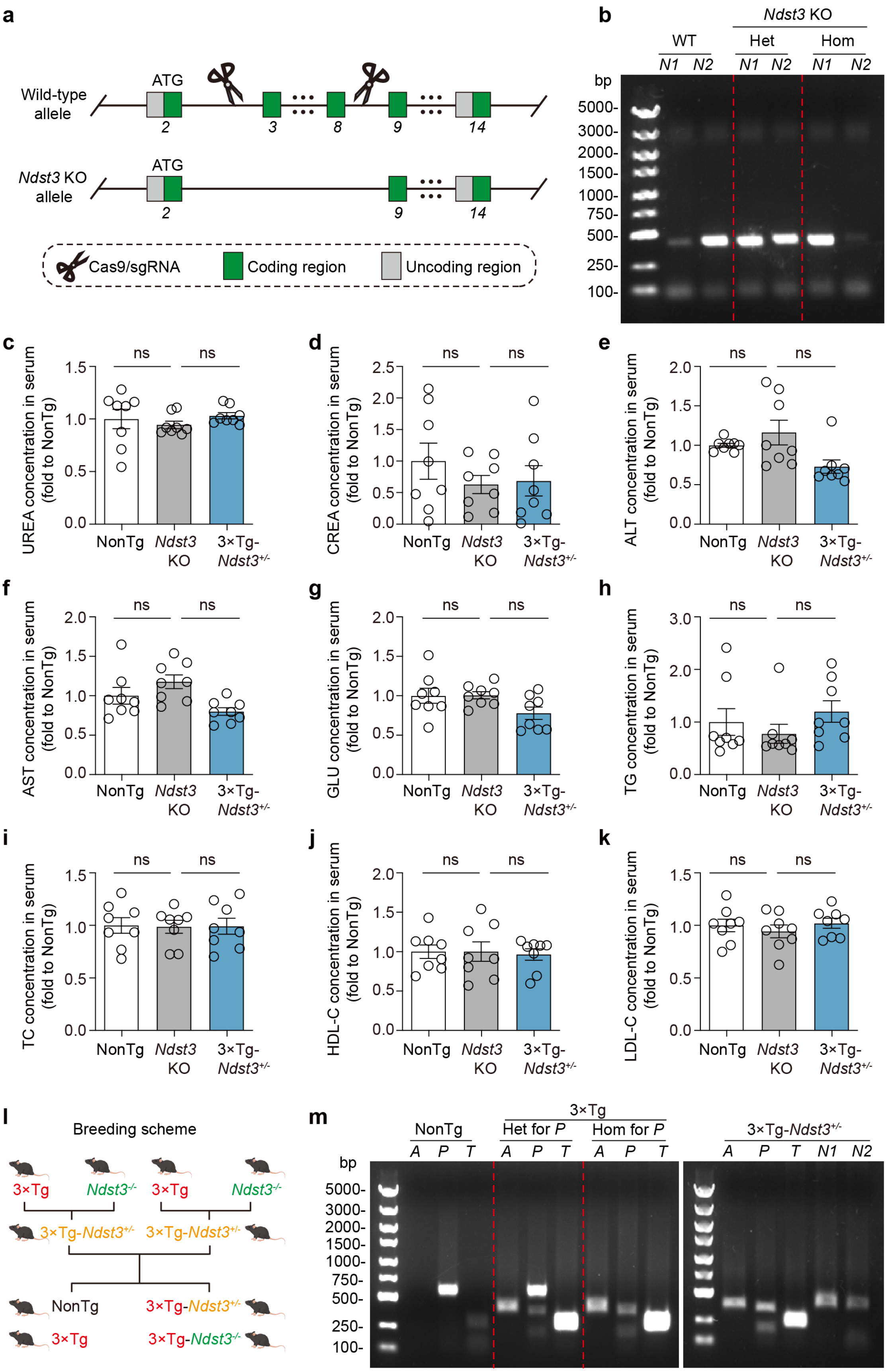
a
Wild-type allele
ATG
2
3
8
9
14
Ndst3 KO allele
ATG
2
9
14
Cas9/sgRNA
Coding region
Uncoding region
b
Ndst3 KO
WT
Het
Hom
N1
N2
N1
N2
N1
N2
bp
5000-
3000-
2000-
1500-
1000-
750-
500-
250-
100-
c
UREA concentration in serum (fold to NonTg)
d
CREA concentration in serum (fold to NonTg)
e
ALT concentration in serum (fold to NonTg)
f
AST concentration in serum (fold to NonTg)
g
GLU concentration in serum (fold to NonTg)
h
TG concentration in serum (fold to NonTg)
i
TC concentration in serum (fold to NonTg)
j
HDL-C concentration in serum (fold to NonTg)
k
LDL-C concentration in serum (fold to NonTg)
ns
NonTg
Ndst3 KO
3×Tg-Ndst3+/-
l
Breeding scheme
3×Tg
Ndst3-/-
3×Tg-Ndst3+/-
NonTg
3×Tg
3×Tg-Ndst3-/-
m
3×Tg
NonTg
Het for P
Hom for P
3×Tg-Ndst3+/-
A
P
T
N1
N2
bp
5000-
3000-
2000-
1500-
1000-
750-
500-
250-
100-

95

Figure S6. *Ndst3* KO and 3xTg-$Ndst3^{+/-}$ mouse generation, genotyping and blood biochemical analyses. **a** The design strategy for *Ndst3* knockout (KO) mouse line. **b** Genotyping of *Ndst3* KO mice. The heterozygous *Ndst3* KO ($Ndst3^{+/-}$) mice were positive for *N1* (402 bp) and *N2* (414 bp), while the homozygous ($Ndst3^{-/-}$) mice were positive for *N1* (402 bp) and the WT mice were positive for *N2* (414 bp). *N1* represents the first pair of *Ndst3* primers; *N2*, the second pair of *Ndst3* primers. **c**-**k** Blood biochemical analyses of serum urea (UREA), creatinine (CREA), alanine transaminase (ALT), aspartate transaminase (AST), glucose (GLU), triglycerides (TG), total cholesterol (TC), high-density lipoprotein cholesterol (HDL-C), and low-density lipoprotein cholesterol (LDL-C) in NonTg, *Ndst3* KO, and 3xTg-$Ndst3^{+/-}$ mice (*n* = 8 mice per group; ns represents nonsignificant). **l** The hybridization strategy for 3xTg-$Ndst3^{+/-}$ mouse line. The mouse in this panel was drawn using Figdraw (https://www.figdraw.com). **m** Genotyping of 3xTg-AD and 3xTg-$Ndst3^{+/-}$ mice. Distinct $PSEN1^{M146V}$ (*P*) banding patterns were observed in heterozygous (530/350/180 bp) and homozygous (350/180 bp) 3xTg-AD mice. All 3xTg-AD mice were positive for $APP695^{Swe}$ (*A*, 377 bp) and $MAPT/tau^{P301L}$ (*T*, 243 bp). NonTg mice exhibited only the 530 bp *P* band. Het represents heterozygous; Hom, homozygous.

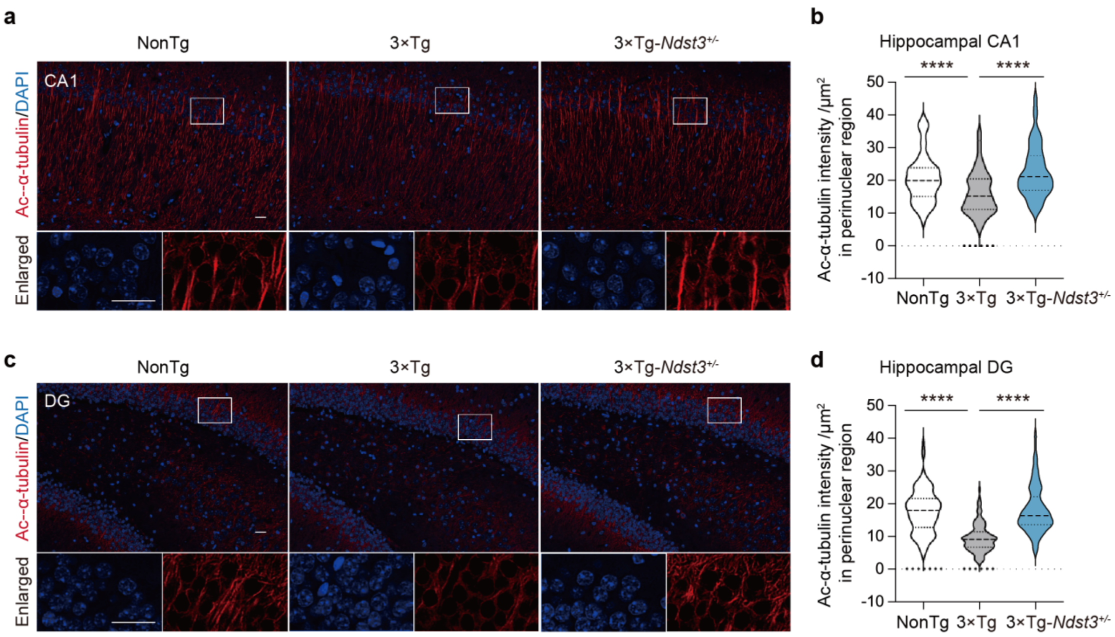

Figure S7. Perinuclear microtubule acetylation in the hippocampi of 10-month-old 3xTg-*Ndst3*$^{+/-}$ mice and age-matched NonTg and 3xTg-AD controls. **a**, **b** Representative immunofluorescence images (**a**) and quantification (**b**) of perinuclear Ac-α-tubulin intensity in the hippocampal CA1 regions of NonTg, 3xTg-AD, and 3xTg-*Ndst3*$^{+/-}$ mice (n = 96 cells from three mice; ****P < 0.0001). **c**, **d** Representative immunofluorescence images (**c**) and quantification (**d**) of perinuclear Ac-α-tubulin intensity in the hippocampal DG regions of NonTg, 3xTg-AD, and 3xTg-*Ndst3*$^{+/-}$ mice (n = 125 cells from three mice; ****P < 0.0001). The perinuclear region was defined as the area within 5 μm of the DAPI-stained cell nucleus. Scale bar, 25 μm.

123

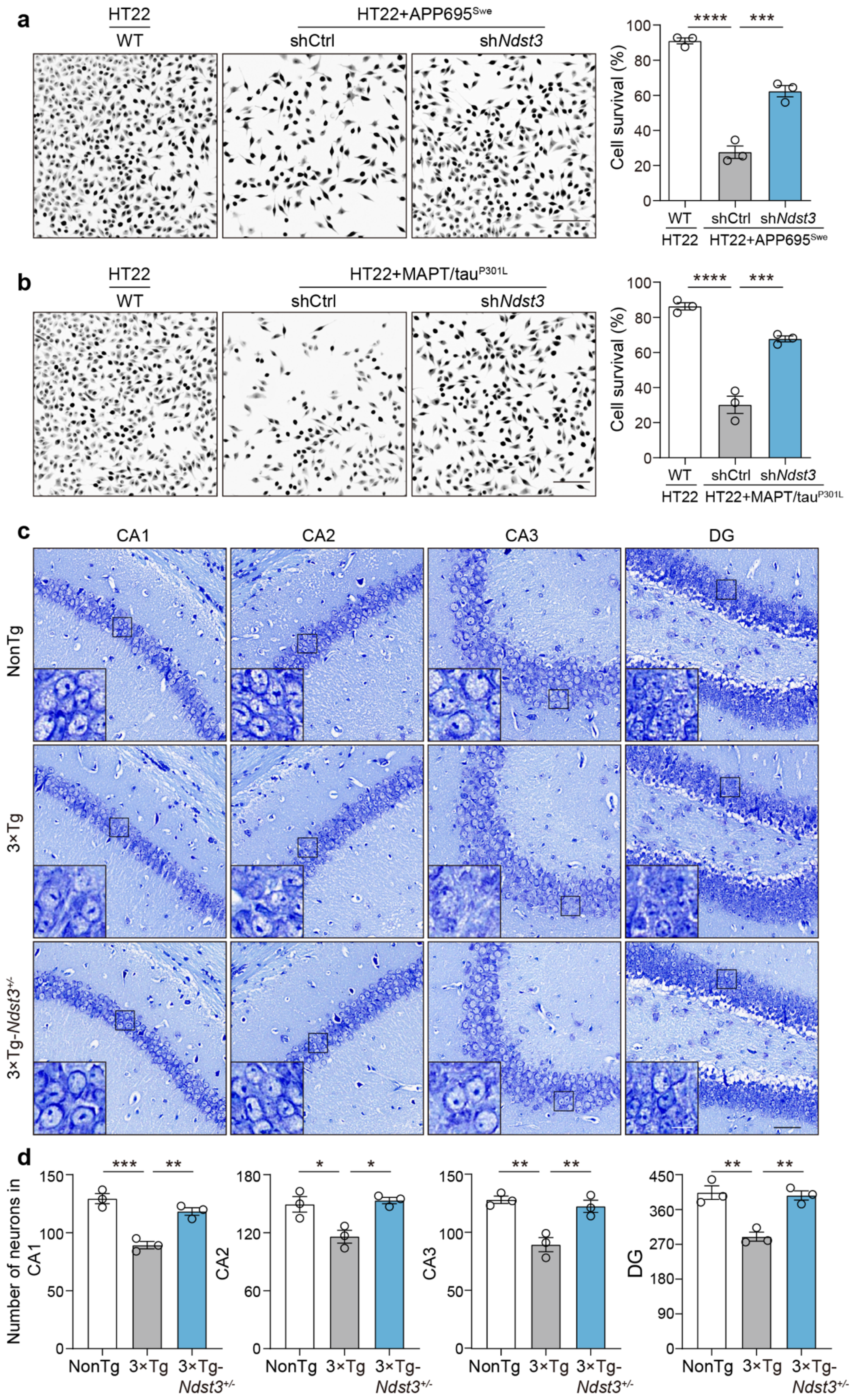
a
HT22
WT
HT22+APP695Swe
shCtrl
shNdst3
Cell survival (%)
100
80
60
40
20
0
****
***
WT
shCtrl
shNdst3
HT22
HT22+APP695Swe
b
HT22
WT
HT22+MAPT/tauP301L
shCtrl
shNdst3
Cell survival (%)
****
***
HT22
HT22+MAPT/tauP301L
c
CA1
CA2
CA3
DG
NonTg
3×Tg
3×Tg-Ndst3+/-
d
Number of neurons in
CA1
CA2
CA3
DG
150
100
50
180
120
60
450
360
270
180
90
***
**
*
NonTg
3×Tg
3×Tg-
Ndst3+/-

Figure S8. Cell survival in APP695^{Swe}- or MAPT/tau^{P301L}-overexpressing HT22 cells with NDST3 knockdown, and Nissl staining in the hippocampus of NonTg, 3xTg, and 3xTg-*Ndst3*$^{+/-}$ mice. **a**, **b** Calcein AM staining was performed to assess the survival percentage of HT22 cells 48 h after they were transduced with APP695^{Swe} (**a**) or MAPT/tau^{P301L} (**b**). HT22 cells transfected with an WT APP695 or WT MAPT/tau control plasmid along with a scrambled shRNA were set as controls. The cell survival was quantified as the percentage of the fluorescence intensity for transfected cells relative to non-transfected positive controls (*n* = 3 independent cultures; ***P <0.001, ****P <0.0001). Scale bar, 100 μm. **c** Nissl staining images of hippocampal CA1, CA2, CA3 and DG in 10-month-old NonTg, 3xTg-AD, and 3xTg-*Ndst3*$^{+/-}$ mice. Scale bar, 50 μm (main panels), 10 μm (insets). **d** Quantitative analysis of the number of normal neurons in the CA1, CA2, CA3, and DG regions of the hippocampus based on the Nissl staining in (**c**) (*n* = 3 mice; *P < 0.05, **P < 0.01, ***P < 0.001). Error bars represent ± SEMs. One-way ANOVA followed by Dunnett's post hoc tests are used for the statistical analyses.

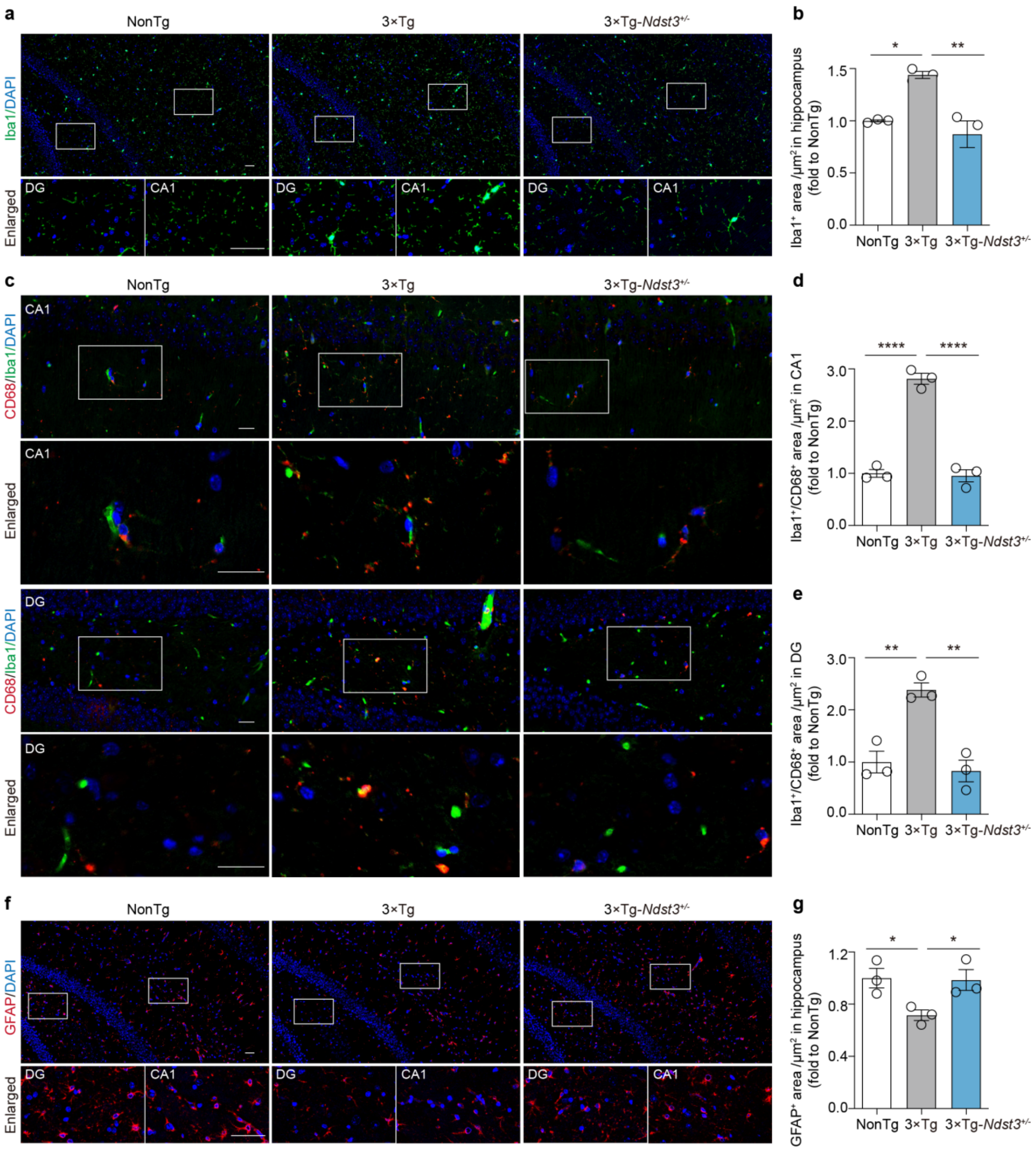

Figure S9. Microglia and astrocyte activation in the hippocampi of 10-month-old NonTg, 3xTg-AD, and 3xTg-*Ndst3*$^{+/-}$ mice. **a**, **b** Representative immunofluorescence images (**a**) and quantification (**b**) of microglia marker Iba1 in the hippocampus of NonTg, 3xTg-AD, and 3xTg-*Ndst3*$^{+/-}$ mice (Scale bar, 50 μm; *n* = 3 mice; **P* = 0.0118, ***P* = 0.0035). **c-e** Representative co-immunofluorescence images (**c**) and colocalization area quantification of Iba1 and activated microglia marker CD68 in hippocampal CA1 (**d**) and DG (**e**) regions of NonTg, 3xTg-AD, and 3xTg-*Ndst3*$^{+/-}$ mice (Scale bar, 25 μm; *n* = 3 mice; ***P* < 0.01, *****P* < 0.0001). **f**, **g** Representative immunofluorescence images (**f**) and quantification (**g**)

of astrocyte marker GFAP in the hippocampus of NonTg, 3xTg-AD, and 3xTg-*Ndst3*$^{+/-}$ mice (Scale bar, 50 μm; $n = 3$ mice; $*P < 0.05$).

Table S1. Primer sequences used for genotyping of *Ndst3* knockout and 3xTg Alzheimer's disease mouse models

Gene	Forward (5'-3')	Reverse (5'-3')
APP695^{Swe}	AGGACTGACCACTCGACCAG	CGGGGGTCTAGTTCTGCAT
PSEN1^{M146V}	AGGCAGGAAGATCACGTGTTCAAGTAC	CACACGCACACTCTGACATGCACAGGC
MAPT/tau^{P301L}	CTTTGAACCAGGATGGCTGA	TCCCGTCTTTGCTTTTACTGA
Ndst3 (N1)	GCAGGCAATCTTCAAACAGTAGC	CCTTCTCAGCATGATCGTACAGTG
Ndst3 (N2)	CTTCCTTGGCTTCCAGTACCTTC	GCACAGTCCTCAGACGACTAGG

Note: *APP695*^{Swe}, Amyloid precursor protein with Swedish mutation; *MAPT/tau*^{P301L}, microtubule-associated protein tau with P301L mutation; *Ndst3*, N-Deacetylase and N-Sulfotransferase 3; *N1*, First pair of primers; *N2*, Second pair of primers; *PSEN1*^{M146V}, Presenilin-1 with M146V mutation.

Table S2. Characteristics of postmortem temporal cortex samples from Alzheimer's disease patients and control subjects

Sample No.	Source	AD pathology	Age of sampling	Gender	Sample type
Con 1	XYSM-HB	No	78	M	Paraffin-embedded temporal cortex
Con 2	XYSM-HB	No	88	M	Paraffin-embedded temporal cortex
Con 3	XYSM-HB	No	67	M	Paraffin-embedded temporal cortex
AD 1	XYSM-HB	Yes	82	M	Paraffin-embedded temporal cortex
AD 2	XYSM-HB	Yes	89	M	Paraffin-embedded temporal cortex
AD 3	XYSM-HB	Yes	70	M	Paraffin-embedded temporal cortex

Note: AD, Alzheimer's disease; Con, Healthy control; M, Male; XYSM-HBB, Human Brain Bank, Central South University Xiangya School of Medicine.
