# Supplementary material for: NDST3 suppression restores lysosomal acidification and ameliorates amyloid-β and MAPT/tau pathology in Alzheimer’s disease
Source: Transl Neurodegener. 2026 Apr 21;15:16. doi: 10.1186/s40035-026-00549-1 (PMC13097816; doi:10.1186/s40035-026-00549-1)
Supplement: Supplementary file 2 — Additional file 2. Uncropped gels and blots. [file 40035_2026_549_MOESM2_ESM.pdf]

Figure 2c

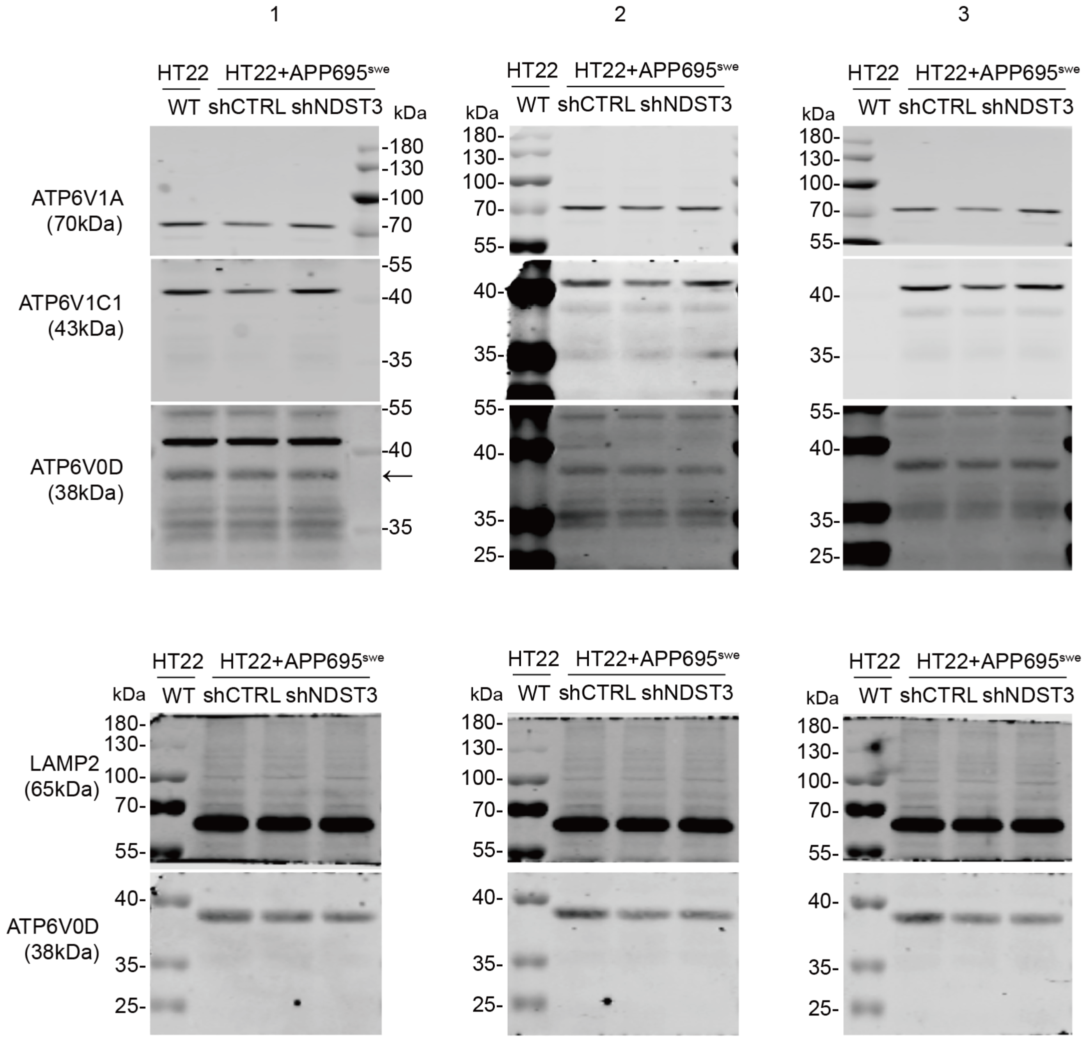

1
2
3
HT22
HT22+APP695swe
WT
shCTRL
shNDST3
kDa
ATP6V1A
(70kDa)
ATP6V1C1
(43kDa)
ATP6V0D
(38kDa)
LAMP2
(65kDa)
180-
130-
100-
70-
55-
40-
35-
25-

Figure 3e

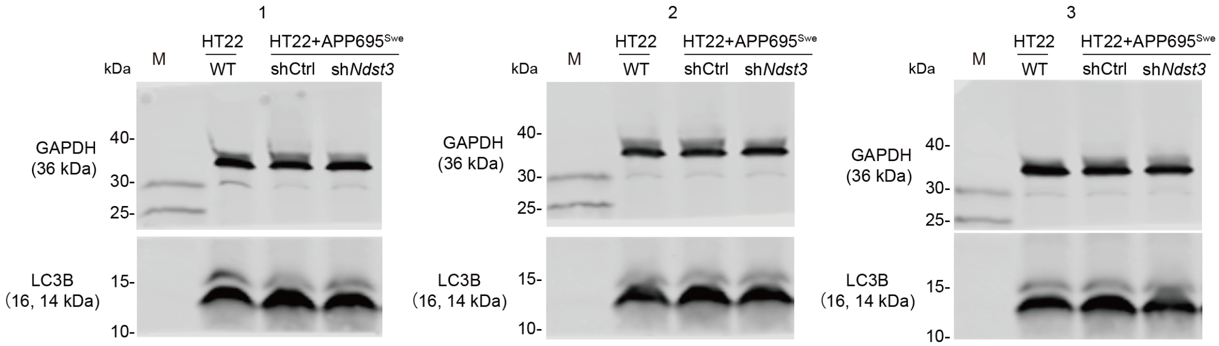

Figure 3g

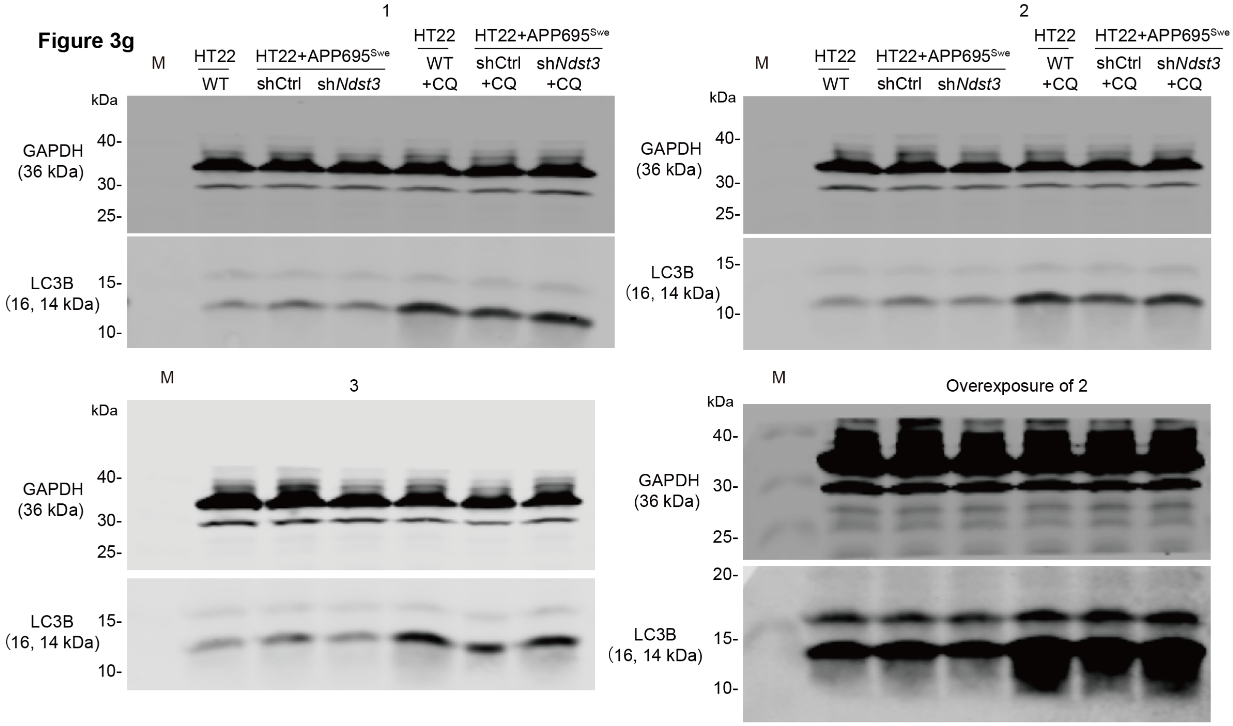

Figure 3k

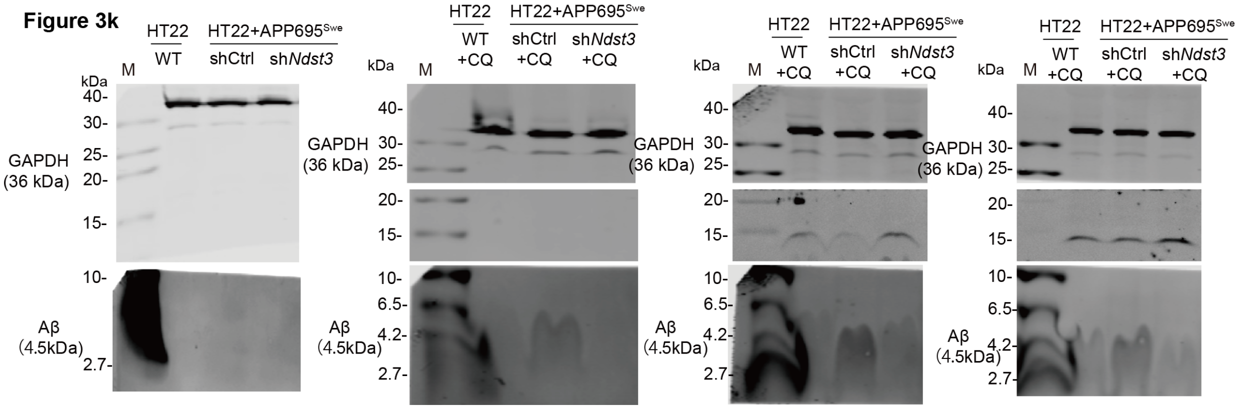

Figure 3m

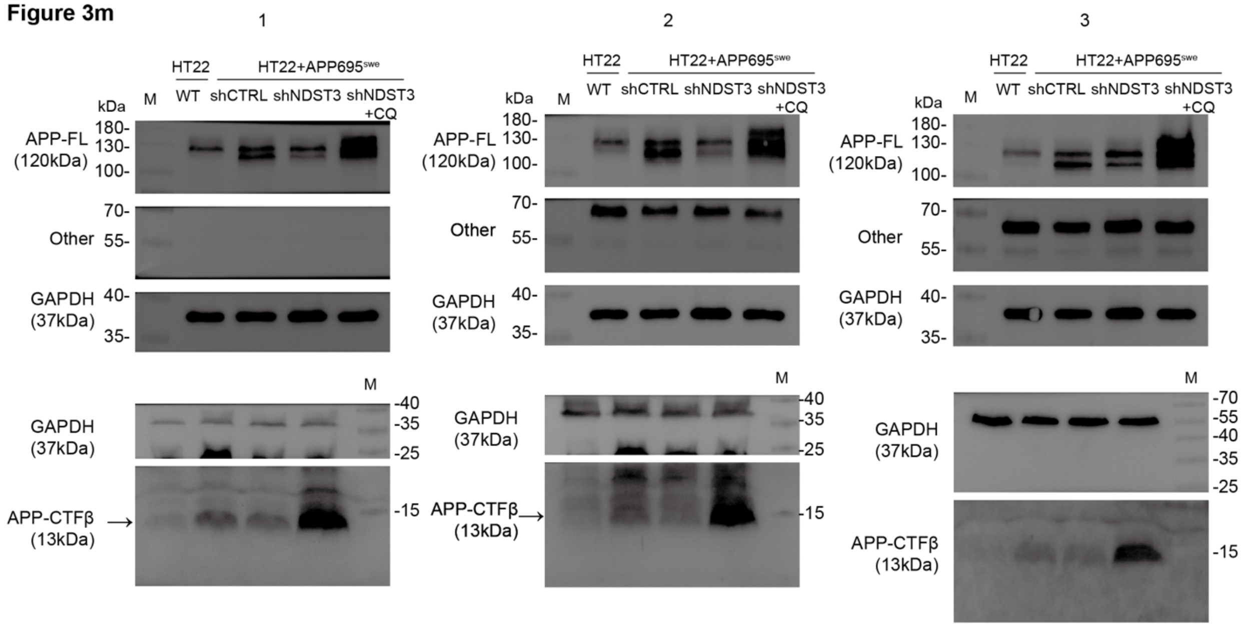

Figure 3q

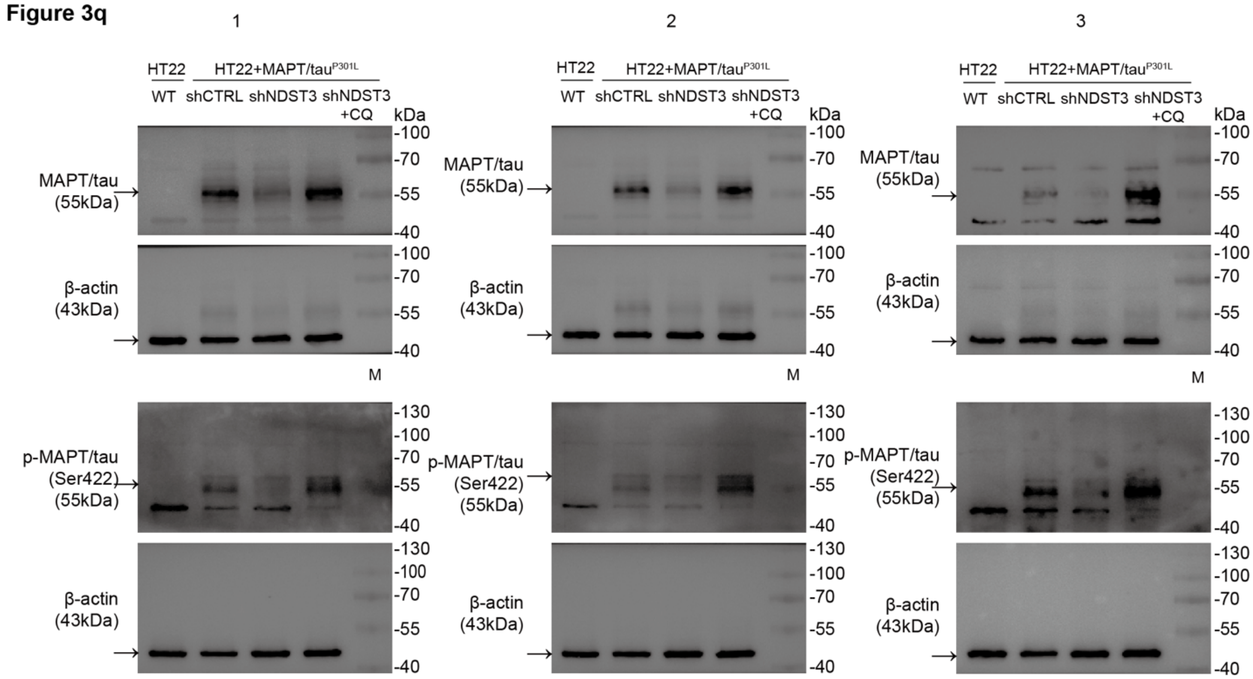

Figure 4a

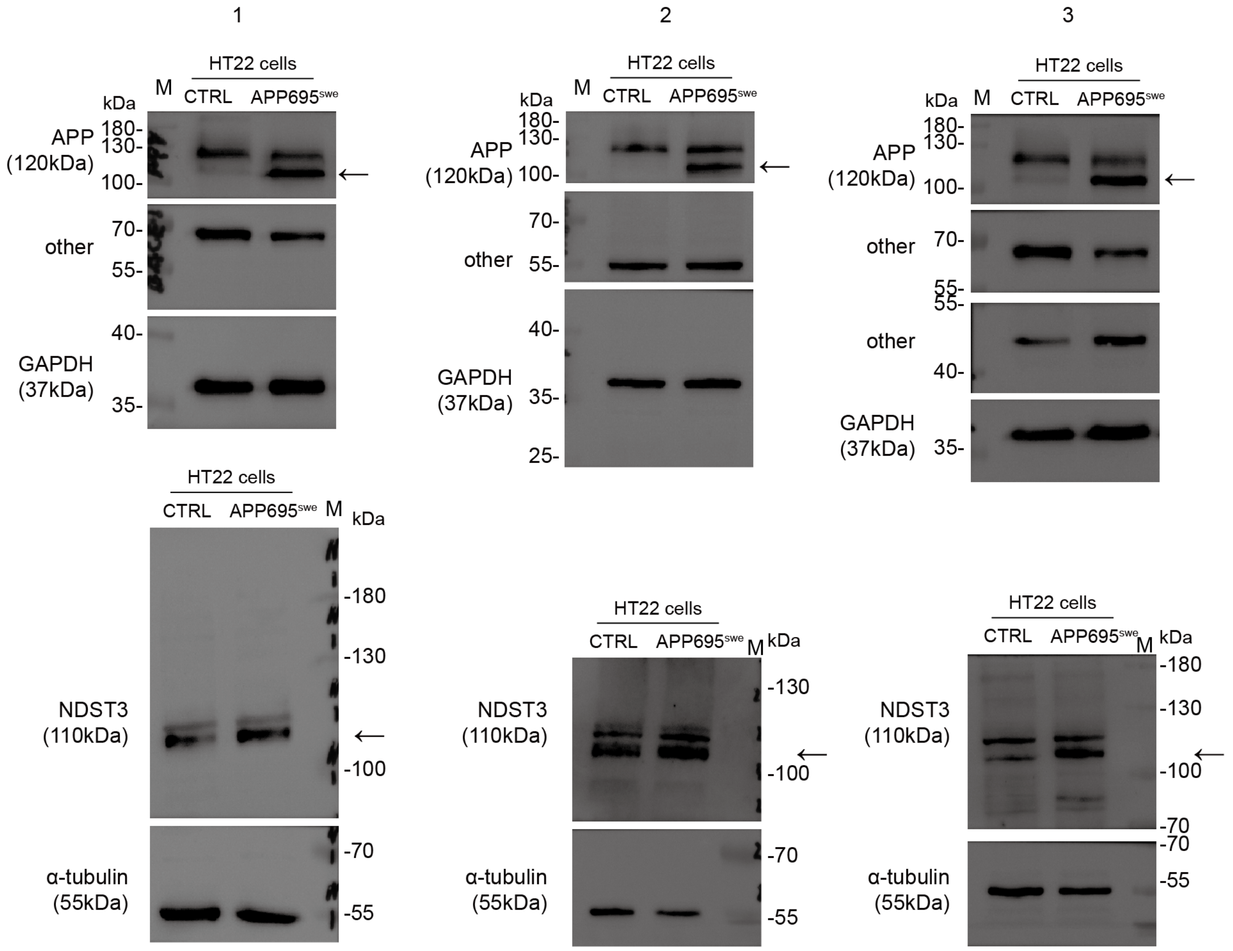

1
2
3
HT22 cells
M
CTRL
APP695swe
kDa
APP (120kDa)
180-
130-
100-
other
70-
55-
GAPDH (37kDa)
40-
35-
25-
NDST3 (110kDa)
-180
-130
-100
-70
α-tubulin (55kDa)
-55

Figure 5a

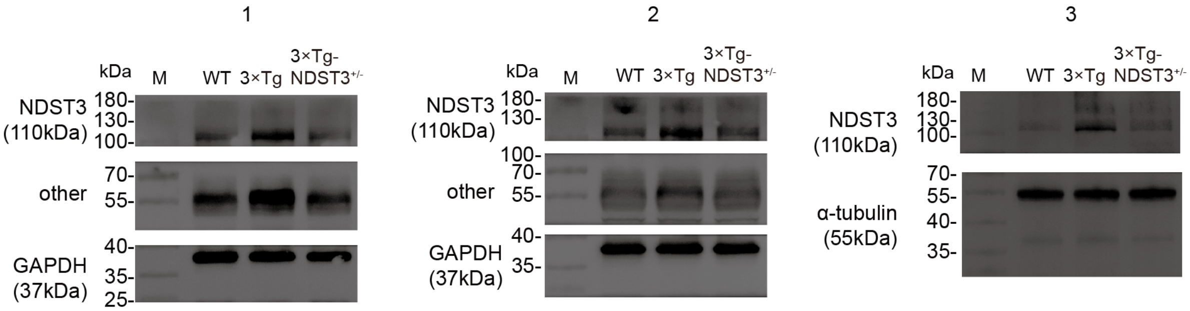

Figure 5e

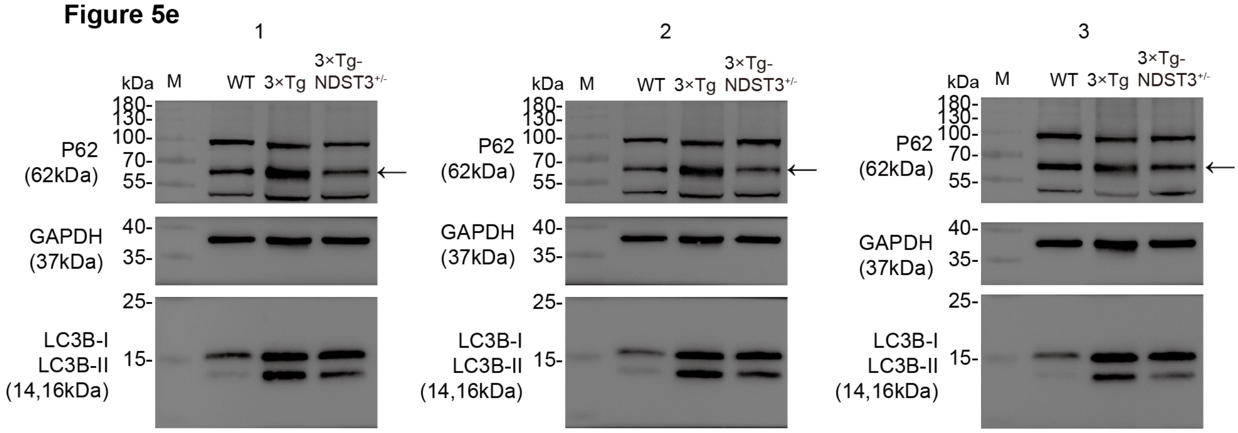

Figure 5h

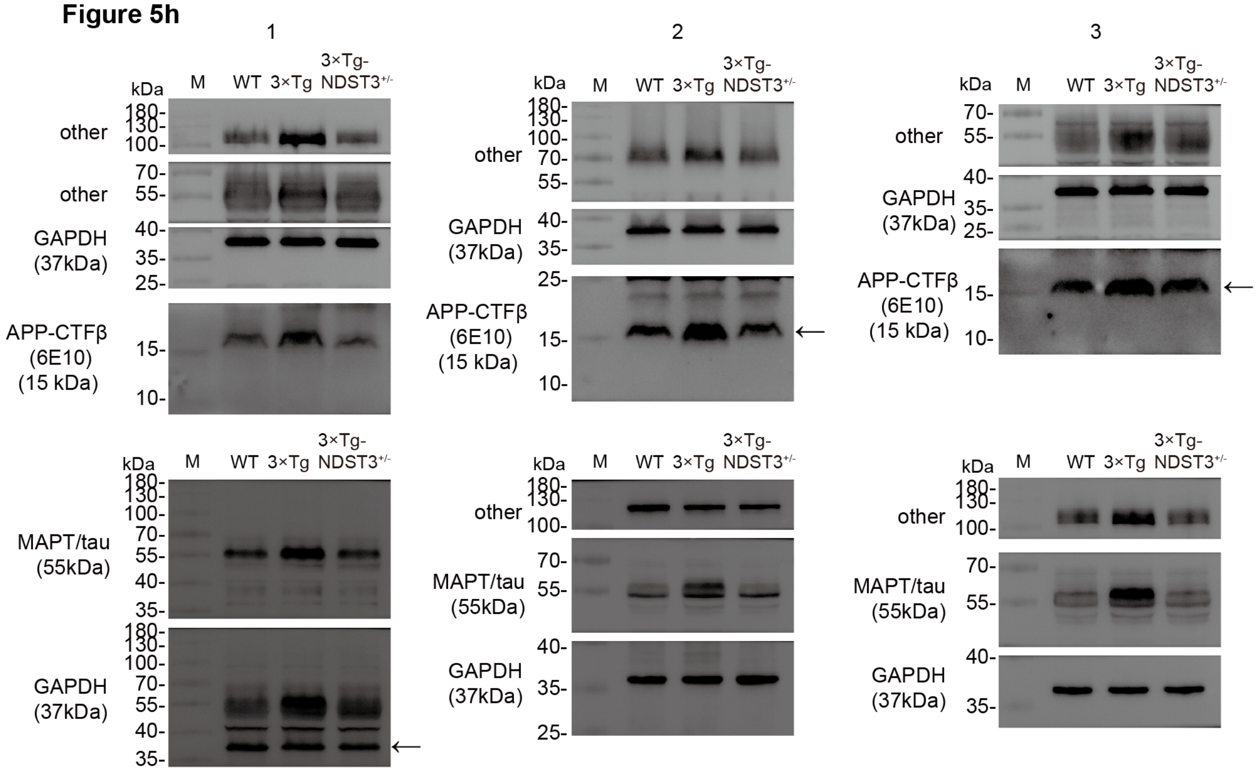

Figure S1e

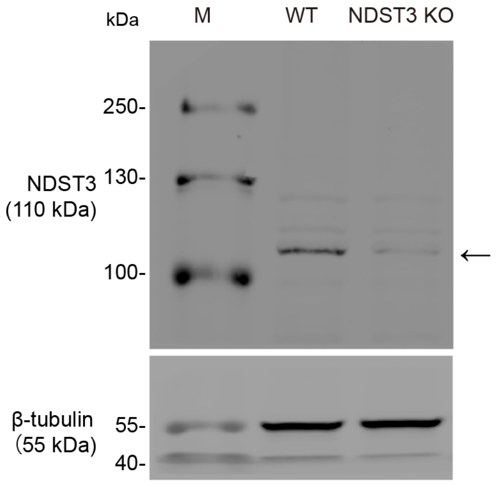

Figure S1f

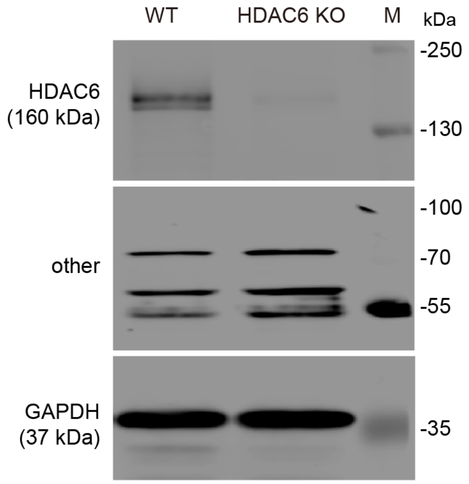

Figure S1i

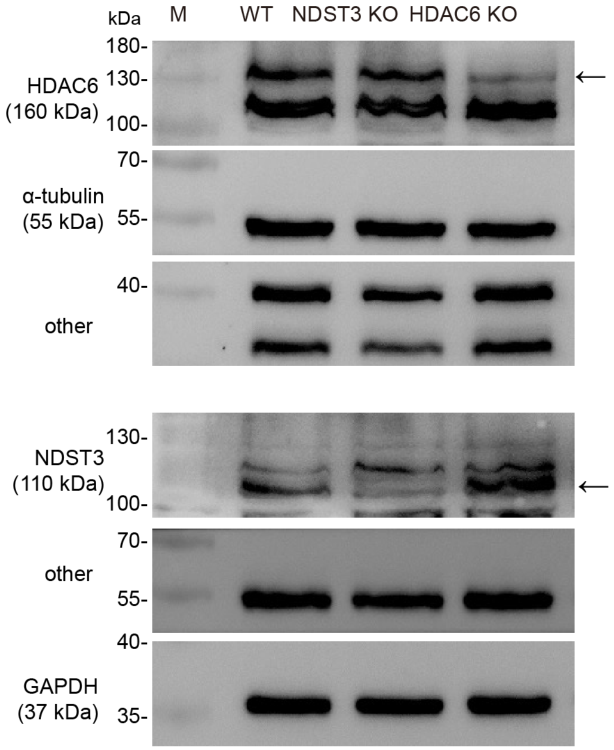

Figure S2a

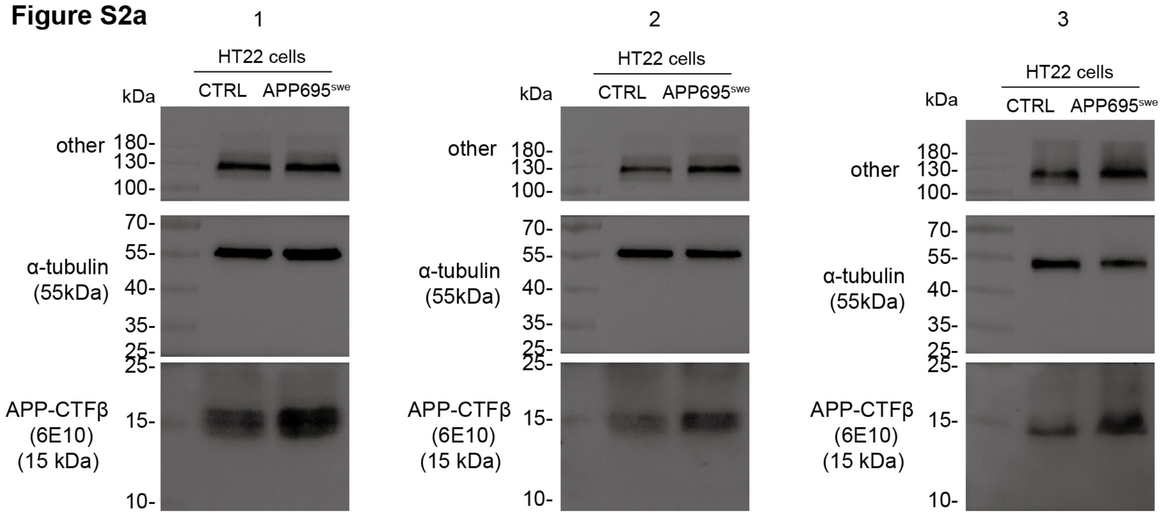

Figure S2e

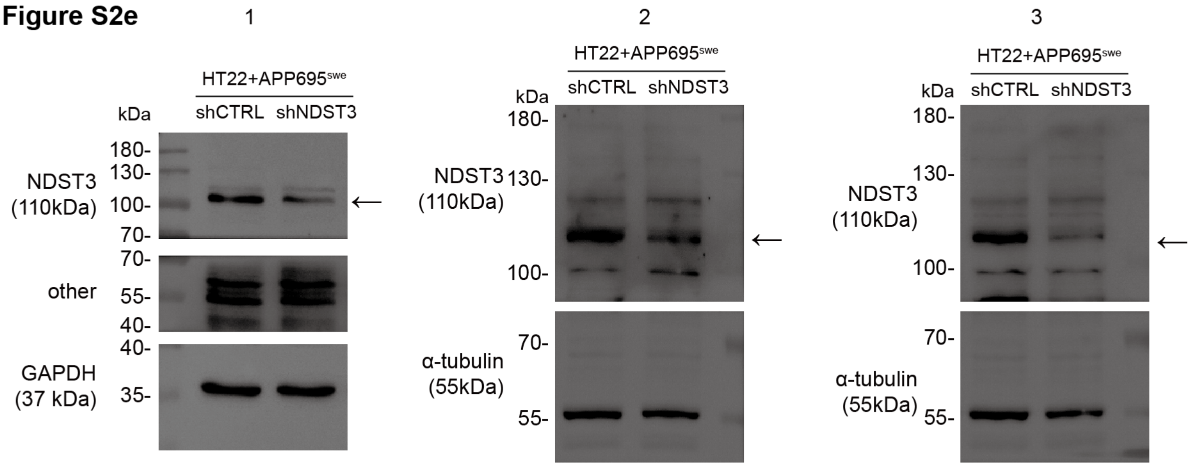

Figure S2g

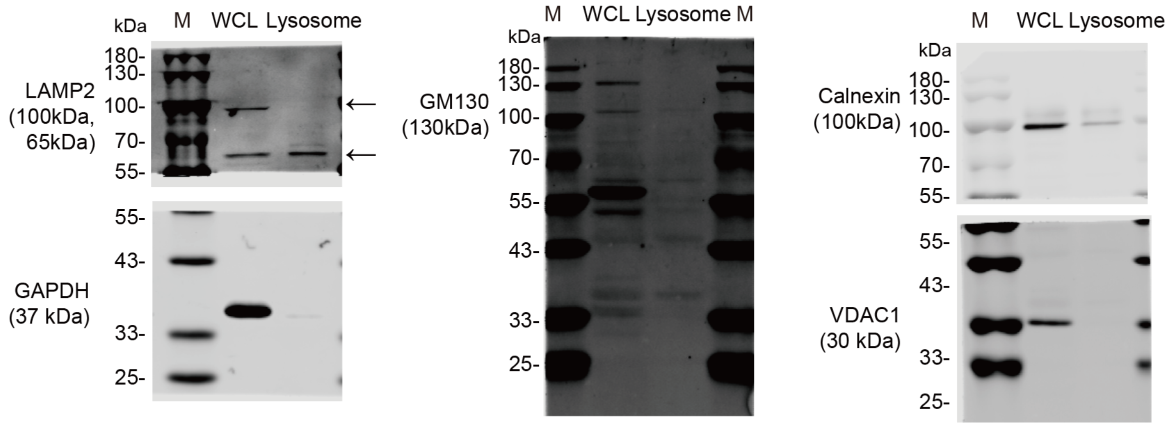

Figure S2j

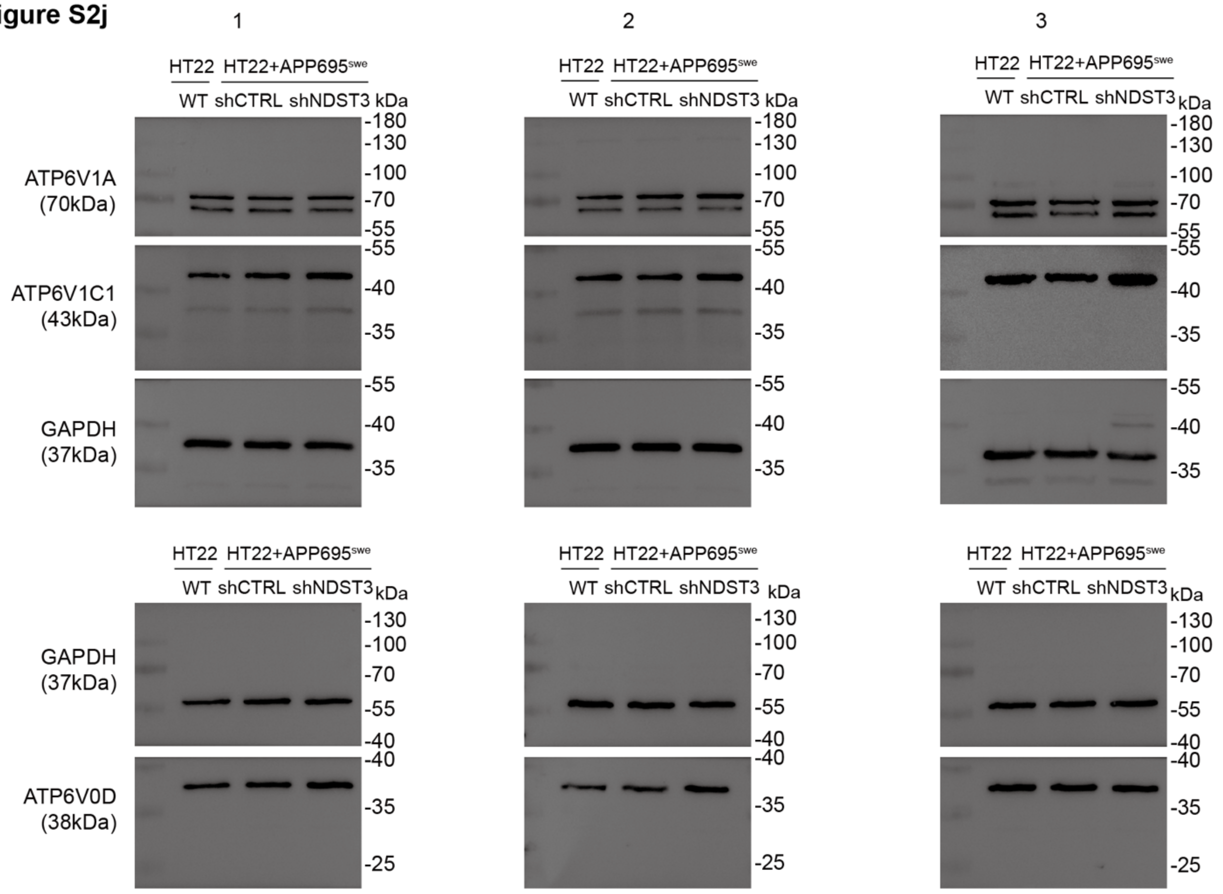

Figure S4a

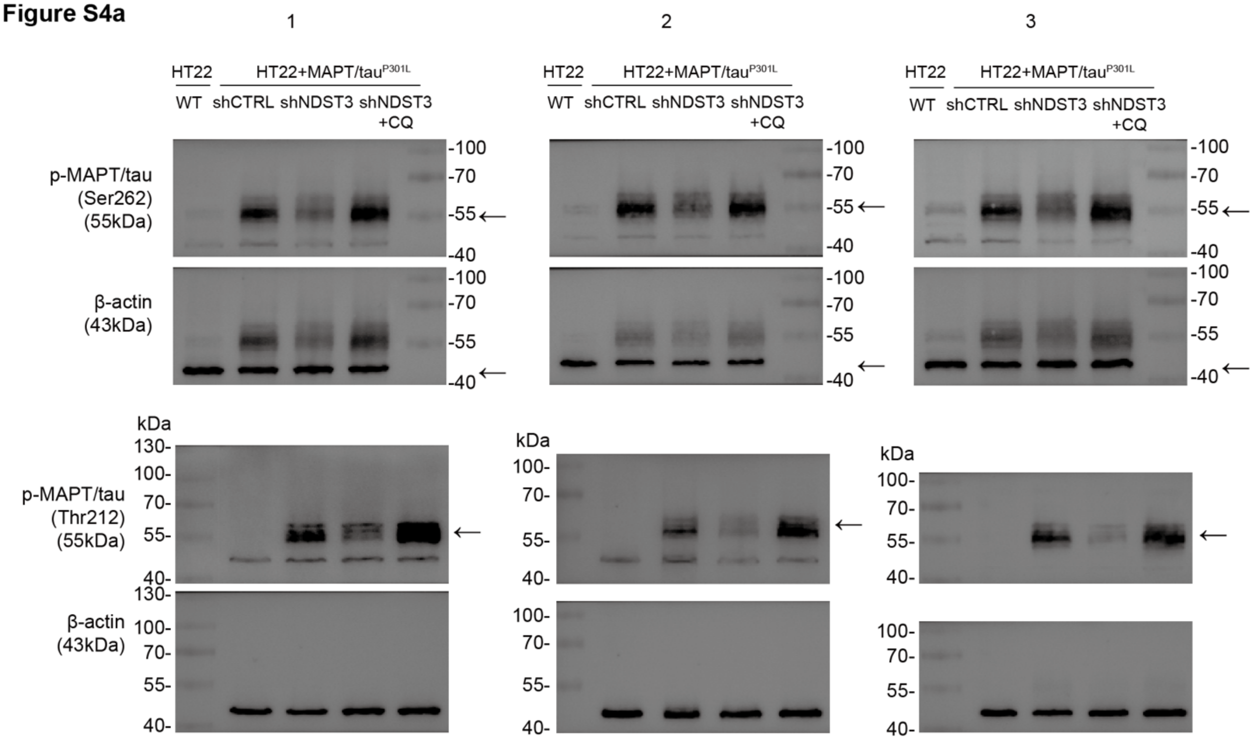

Figure S5g

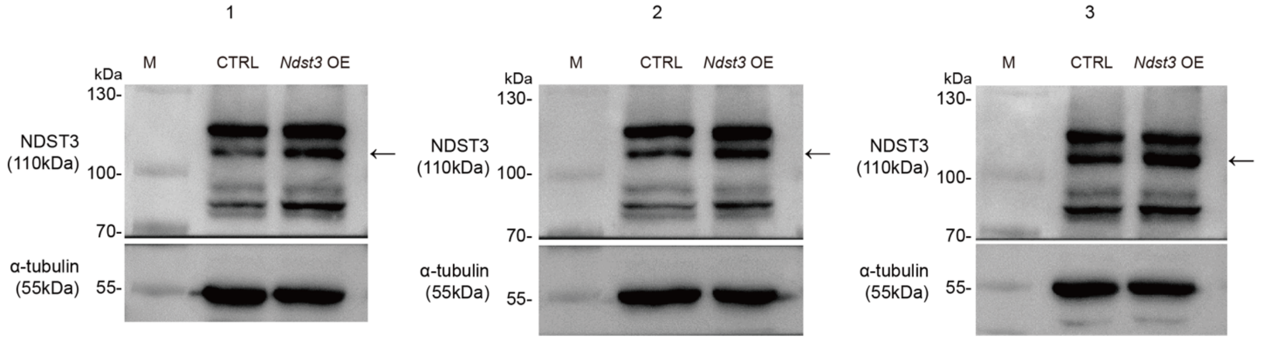
1
2
3
M
CTRL
Ndst3 OE
kDa
130-
100-
70-
55-
NDST3
(110kDa)
α-tubulin
(55kDa)
